# Europium Luminescence: Electronic Densities and Superdelocalizabilities for a Unique Adjustment of Theoretical Intensity Parameters

**DOI:** 10.1038/srep13695

**Published:** 2015-09-02

**Authors:** José Diogo L. Dutra, Nathalia B. D. Lima, Ricardo O. Freire, Alfredo M. Simas

**Affiliations:** 1 Pople Computational Chemistry Laboratory, Departamento de Química, CCET, UFS, 49100-000, Aracaju, SE, Brazil; 2 Departamento de Química Fundamental, CCEN, UFPE, 50590-470, Recife, PE, Brazil

## Abstract

We advance the concept that the charge factors of the simple overlap model and the polarizabilities of Judd-Ofelt theory for the luminescence of europium complexes can be effectively and uniquely modeled by perturbation theory on the semiempirical electronic wave function of the complex. With only three adjustable constants, we introduce expressions that relate: (i) the charge factors to electronic densities, and (ii) the polarizabilities to superdelocalizabilities that we derived specifically for this purpose. The three constants are then adjusted iteratively until the calculated intensity parameters, corresponding to the ^5^*D*_0_→^7^*F*_2_ and ^5^*D*_0_→^7^*F*_4_ transitions, converge to the experimentally determined ones. This adjustment yields a single unique set of only three constants per complex and semiempirical model used. From these constants, we then define a binary outcome acceptance attribute for the adjustment, and show that when the adjustment is acceptable, the predicted geometry is, in average, closer to the experimental one. An important consequence is that the terms of the intensity parameters related to dynamic coupling and electric dipole mechanisms will be unique. Hence, the important energy transfer rates will also be unique, leading to a single predicted intensity parameter for the ^5^*D*_0_→^7^*F*_6_ transition.

The theoretical foundation for the first lanthanide luminescence models began to burgeon in the late ′20 s. Through the Point Charge Electrostatic Model, PCEM, Bethe estimated the magnitude of the crystalline electric field on the energy levels of the 4d and 4f orbitals^1^. In 1937, Van Vleck assigned the narrow spectral lines observed for the lanthanide ions to 4f transitions. Further, in this same article, Van Vleck addressed the nature of these electronic transitions and classified them as governed by electric dipole, magnetic dipole and electric quadrupole mechanisms^2^. Furthermore, eight years later, by semi-quantitative calculations, Broer and coauthors demonstrated that the electric dipole mechanism was sufficient to explain the observed experimental intensities^3^.

These were the works that inspired and gave support to Judd-Ofelt theory^4^,^5^. Indeed, in 1962, Judd^4^ and Ofelt^5^ published, in an independent manner, their studies on the transitions between the electronic energy levels in the 4f sub-shell of lanthanide ions. In their articles, they both formulated essentially the same theory that quantitatively explains the radiative optical transitions in the lanthanide ions, in which they used Racah algebra to arrive at expressions for the oscillator strengths related to the forced electric dipole terms within 4f^n^ configurations^4^,^5^. The calculation of intensity parameters through the Judd-Ofelt theory depends on the contributions of two important terms that represent two mechanisms, each: (i) the forced electric dipole mechanism, and (ii) the dynamic coupling mechanism.

Calculation of the forced electric dipole mechanism depends on the odd parity crystal field parameters. Until the early 80 s, these parameters were obtained from PCEM. However, at that time, reports in the literature suggested that the even-parity terms obtained by PCEM do not correspond to the observed crystal field splitting in lanthanide ions^6^,^7^,^8^. To overcome part of these discrepancies, in 1982, Malta introduced the Simple Overlap Model—SOM^9^. The SOM model assumes two postulates: (i) the 4f energy potential is generated by charges, uniformly distributed in a small region located around the midpoints of the lanthanide–ligand chemical bonds, and (ii) the total charge in each region is equal to -*geρ*, where *g* is a charge factor, *e* is the fundamental electric charge, and *ρ* is a parameter proportional to the magnitude of the total overlap between the lanthanide ion and the ligand atoms.

While PCEM only treats the metal-ligand atom bonds as a purely electrostatic phenomenon, SOM introduces a correction to the crystal field parameters of PCEM in order to confer to it a degree of covalency through a charge factor *g*. However, the SOM article^9^ did not provide equations for its calculation.

The calculation of intensity parameters through the Judd-Ofelt theory further depends on equations describing the dynamic coupling mechanism, which in turn depends on structural aspects (coordination geometry), and is thus sensitive to the chemical environment around the lanthanide ion through polarizabilities, α_i_, of the i directly coordinated atoms of the ligands^10^. So far, there are no expressions that allow the calculation of these polarizabilities. More recently, Malta and co-workers introduced the concept of the overlap polarizability of a chemical bond and proposed an ordinal scale of covalence for lanthanide complexes^11^. They also proposed an equation for calculating this new overlap polarizability^11^. However, this overlap polarizability is only a part of the polarizability itself, i.e. this equation does not fully quantify the polarizabilities α_i_.

Hence, to this day, the charge factors of the SOM model and the polarizabilities of the Judd-Ofelt theory do not have any mathematical expressions to allow them to be evaluated.

In 1994, we introduced the Sparkle Model to carry out semiempirical molecular orbital calculation of lanthanide complexes at the AM1 level^12^,^13^, making it also possible to calculate UV-Vis absorption spectra from the Sparkle Model geometry via INDO/S^14^. The model was improved in 2004^15^ with the addition of Gaussian functions to the core-core repulsion and proved useful for the design of luminescent complexes^16^,^17^. Subsequently, robust statistical methodologies were incorporated into the parameterization procedure, and the model has been since parameterized for a variety of existing and widely distributed semiempirical models, such as AM1^18^, PM3^19^,^20^, PM6^21^, PM7^22^, and RM1^23^. We designed the Sparkle Models^24^,^25^,^26^,^27^,^28^ to predict mainly geometries—the most computing time intensive part of lanthanide complex computational chemistry. Indeed, once one has a fully optimized geometry, more advanced single point calculations on the complexes can then be carried out with more workability. The variety of Sparkle Model^24^,^25^,^26^,^27^,^28^ implementations is important because ligands in the complexes vary, and different semiempirical models tackle particular bonding situations differently: some more accurately than others. Therefore, having a palette of Sparkle Models to choose from, adds a strong value to the experimentalist. All are fully available in the MOPAC software^29^.

Recently, the RM1 model for lanthanides has been introduced^30^. In this model, the europium atom is now represented in the semiempirical calculation as an atom with a core depicting [Xe4f^6^]; while assigning to its semiempirical valence shell, three electrons and the following set of semiempirical atomic orbitals: 5d 6s 6p. The RM1 model for lanthanides so defined, does extend the accuracy of the previous Sparkle Models to types of coordinating bonds other than Eu-O and Eu-N; the most common ones for Eu being Eu-C, Eu-S, Eu-Cl, and Eu-Br.

Both the Sparkle Model and the RM1 model for the lanthanides are quantum chemical models, which generate electronic wave functions, and therefore yield a wealth of information. However, it is noteworthy that, up to now, the electronic wave functions of these models have not been directly used in the context of lanthanide luminescence.

Indeed, the last 20 years were fraught with publications about the development and application of theoretical methods to study the luminescent properties of lanthanide compounds, especially for systems containing europium ions. However, even now, less than 3% of published studies involving lanthanide ions make use of theoretical tools^31^.

In 2013, in order to better disseminate these theoretical tools, our group published an article showing, systematically, the theoretical study of a simple system of europium^32^, and then released our new luminescence software package, LUMPAC^31^. This is the first and only software specifically designed for the study of luminescence properties of systems containing lanthanide ions. This first version, which is available via our homepage (www.lumpac.pro.br), was designed to be efficient and user-friendly. In the short time that it has been available, it is already in use by many experimental groups worldwide.

So far, in the first version of LUMPAC^31^ and other articles^33^,^34^,^35^,^36^,^37^,^38^,^39^, the charge factors *g*_i_ and polarizabilities α_i_ are frequently adjusted in order to reproduce the experimental intensity parameters 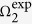 and 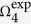. During the adjustment procedure, the calculated intensity parameters (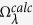) from the optimized geometry, obtained from one of our Sparkle Models, are compared with the experimental intensity parameters (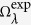).

In this article, we advance the concept that the charges, *g*_i,_ and polarizabilities, α_i_, for europium complexes, within SOM and Judd-Ofelt theory, can be effective and uniquely modeled by energy variations resulting from perturbations on the semiempirical electronic wave function of the complex. In our conceptualization, the charges will be determined from first order perturbation theory, and the polarizabilities from second order perturbation theory.

## Results

### Uniqueness of g_i_ and α_i_

First, we carried out a series of tests to determine the uniqueness of the adjusted set of parameters *g*_i_ and α_i_ for some representative complexes. We found out numerically that the number of degrees of freedom is actually smaller than the theoretical maximum of 2N_c_, where N_c_ is the coordination number of the complex, due to restrictions that result from the need to accommodate the geometry and the values of 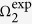 and 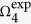 in *g*_i_ and α_i_. Nevertheless, the residual number of degrees of freedom is still quite large. Indeed, there is an enormous space of solutions for this problem, with drastically different values of *g*_i_ and α_i_ leading exactly to the same experimental values of both 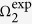 and 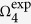. That was an unsettling finding because the different *g*_i_ and α_i_ imply in different predicted 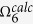 values, on which the radiative emission rate depends. Further, the contribution to the intensity parameters from coupling dynamics (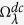), which depends on α_i_, and from electric dipole (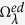), which depend on g_i_, will also vary and be non-unique for any geometry and any two given values of 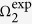 and 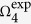. Furthermore, 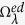, is used to predict the energy transfer rates via the multipolar mechanism, which will, in turn, be non-unique, depending on the *g*_i_ and α_i_ values chosen from the space of solutions to the problem of finding a set of *g*_i_ and α_i_ consistent with the two values 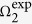 and 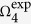.

So, in this article, we introduce a way of determining the sets of *g*_i_ and α_i_ in a unique manner for any given complex, from which the geometry and the values of 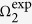 and 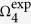 are known.

### Determining *g*_*i*_ and *α*_*i*_ uniquely from semiempirical calculations

In order to model the effect by the metal ion on the directly coordinated atoms of the ligands, we use first and second order perturbations on the semiempirical wavefunction^40^, as fully described in the Supplementary Information which accompanies this article.

Accordingly, in this article, we postulate that the charge factors *g*_*i*_ of the SOM model^41^ are equal to the following expression obtained from first order perturbation theory:


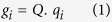


where *Q* will be a single parameter to be applied to all zero differential overlap, ZDO, electronic densities, *q*_*i*_, of all directly coordinated atoms i. The expression for the ZDO electronic density at any atom μ of the complex, q_μ_ is


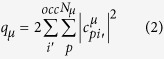


where i’ runs over all occupied molecular orbitals of the complex, p runs over all atomic orbitals of atom μ, and 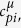 is the corresponding linear coefficient.

Likewise, we further postulate that the polarizabilities α_i_ of Judd-Ofelt theory^4^,^5^ are given by:


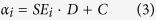


obtained from second order perturbation theory, with constants *D* and *C* being the same for all directly coordinated atoms *i* of a given complex, and *SE*_*σ*_ is the electrophilic superdelocalizability of any atom *σ* of the complex, originally introduced by Simas^40^,


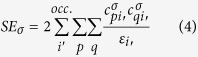


where i’ runs over all occupied molecular orbitals of the complex, p and q run over all atomic orbitals of atom μ, and 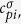 and 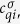 are the corresponding linear coefficients. Our electrophilic superdelocalizabilty is therefore a generalization to an all valence electron method of the corresponding superdelocalizability of Fukui^42^.

In this sense, our electrophilic superdelocalizability^40^ is unique and differs from the one in the article by Lewis^43^ and also from the one in the article by Brown and Simas^44^ because these do not take into account the cross-products of the atomic orbitals for each molecular orbital. And it also differs even more from the delocalizability of Schüürmann^45^,^46^, *D*^*E*^(*i*), because not only, as Lewis^43^ and as Simas and Brown^44^, he does not take into account the cross-products of the atomic orbitals for each molecular orbital, but also because, instead, he uses a different denominator (see Eq. S56 of the Supplementary Information).

As we carried out research for this article, we also tried to use the superdelocalizability of Lewis^43^ and of Simas and Brown^44^, and also the delocalizability of Schüürmann in Eq. (58). However, they all did not produce good fittings. Therefore, we stayed with our superdelocalizability as defined by Eq. (4) above.

Complete derivations of first order and second order perturbations on the semiempirical wavefunction of the lanthanide complex, leading to the ZDO electronic densities, to the electrophilic superdelocalizabilities Eq. (4), and to Eqs. (1) and (3), are fully presented in the Supplementary Information.

Parameters *Q*, *D*, and *C* are then adjusted for each complex in order to reproduce the various experimentally obtained 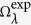 with λ = 2, 4. And in the process of finding the optimal *Q*, *D*, and *C* parameters, we use the nonlinear optimization technique generalized simulated annealing^47^.

### LUMPAC implementation

Both the charge factors from SOM^41^ and the polarizabilities from Judd-Ofelt theory^4^,^5^ introduced by Jørgensen and Judd^10^, to be used in Eqs (S10,S14), must be positive. Since the charge factors, as advanced in the present article, are being calculated from Eq. (1) as a product between the always-positive ZDO electronic densities at the directly coordinated atoms, and a multiplicative constant Q, then this constant Q must always be positive as well.

Likewise, as the electrophilic superdelocalizability is always a negative quantity, and the polarizability must be a positive quantity. Then, from Eq. (3), *SE*_*i*_·*D*+*C* > 0 and therefore, the following inequality must be always obeyed: *C* > −*SE*_*i*_·*D*.

After a large number of simulations, we noted that the *D* and *C* parameters optimized to reproduce the experimental intensity parameters were almost always positive values. Therefore, we restricted the acceptable values of *D* and *C* to positive ones. Further justification of that can be arrived at, by comparing the homomorphic equations Eq. (3) and Eq. (S54) of the Supplementary Information, when it becomes clear that constant *D* can be interpreted as being (*δβ*_*στ*_)^2^ of Eq. (S54), necessarily a positive value. Since *SE*_*i*_ is negative because of the occupied orbital energies in its denominator, then the product *SE*_*i*_·*D* is a negative quantity. As such, the polarizability can only be turned positive by a positive *C*.

Moreover, we also noted that, very frequently, the optimized values of *D* and *C* obeyed approximately the following rule: *D* ≈ *2C*, the vast majority with values of *D* lying in the interval 1 < *D* < 2.5*C*. For the cases where this ratio fell outside this range, the *D/C* ratio found by the non-linear optimization techniques tended to be invariably too small, close to zero. In these cases, the adjustment procedure has not usually been successful. Indeed, when *D* ≈ 0 ≪ *C*, then the effect of the superdelocalizabilities on the polarizabilities is being zeroed and the polarizabilities of all atoms become essentially similar and approximately equal to *C*. In these cases, we regard such fittings to be devoid of physical meaning and discard them. So, there must be some truth in the fact that acceptable fittings always display a *D/C* ratio ≈2 au^−1^, which adds to the strength of the methodology we are introducing in this article. We avail ourselves of this fact and define here a binary outcome acceptance attribute for the adjustment, that is: we consider the adjustment acceptable whenever *D/C* > 1 au^−1^, and unacceptable whenever *D/C* ≤1 au^−1^.

Accordingly, as starting guesses for the parameters in the non-linear optimization of Eq. (5), we then choose values subjected to the conditions *Q* > 0, *D* > 0, *C*> − *SE*_*i*_·*D*, and *D* ≈ *2C*.


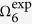
 is rarely observed from emission spectra because it is always displaced towards longer wavelengths and it is also very weak. Thus, in the process of obtaining the fit, we chose to minimize the quadratic errors in 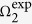 and 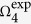, while simultaneously minimizing 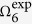 according to Eq. (5), below.


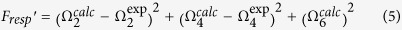


## Discussion

We decided to test the methodology advanced in this article on all europium complexes whose crystallographic structures could be obtained from the Cambridge Crystallographic Database^48^,^49^,^50^, and whose values of 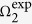 and 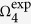 have been published. Thirteen very different complexes obeying this criterion were found, and are listed in Table 1.

**Figure 1:**
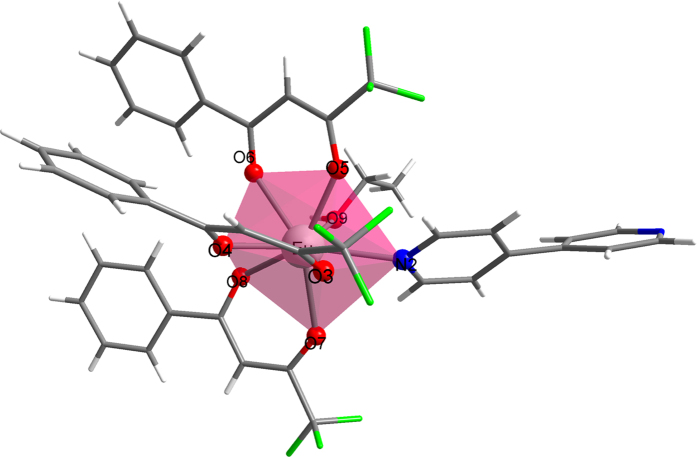
Perspective view of the crystallographic geometry of complex Eu(BTFA)3(4,4-BPY)(EtOH), GIPCAK. Red spheres represent oxygen atoms, blue spheres represent nitrogen atoms, and green sticks represent fluorine. The largest sphere in the center represents the europium atom.

To exemplify how the new methodology functions, consider the crystallographic structure of the complex of CSD code GIPCAK, Eu(BTFA)_3_(4,4-BPY)(EtOH), shown in Fig. 1, where BTFA stands for 4,4,4-trifluoro-1-phenyl-2,4-butanedione, and 4,4-BPY for 4,4′-bipyridine. We then carried out a single point Sparkle/RM1 calculation in order to obtain the ZDO electronic densities and electrophilic superdelocalizabilities at the directly coordinating atoms of the ligands to be used in the fitting procedure. We also carried out single point RM1 model for Eu(III) calculations to obtain the ZDO electronic densities and electrophilic superdelocalizabilities, so that we can now compare the electronic properties results, at the crystallographic geometry, from a Sparkle Model with those from the RM1 model for Eu(III), which has valence orbitals at the europium ion center.

Results are presented in Table 2, which shows the values for *Q*, *D*, and *C* for both Sparkle/RM1 and RM1 model for europium calculations, together with values, at the directly coordinated atoms of the ligands, of ZDO electron densities, electrophilic superdelocalizabilities, and the corresponding charge factors *g* and polarizability α values. Note that the ratio *D/C* is 2.13 au^−1^ for the Sparkle/RM1 case and 2.43 au^−1^ for the RM1 for Eu(III) case.

Observe that the present fitting naturally groups the polarizabilities of the directly coordinated atoms in same ligand groups. That is, the oxygen atoms from one of the BTFAs, BTFA3, have similar Sparkle/RM1 polarizabilities of 6.78 Å^3^ and 6.87 Å^3^; the oxygen atoms from BTFA2 also have similar polarizabilities of 1.30 Å^3^ and 1.23 Å^3^; and for polarizabilities of the oxygen atoms of BTFA1 the values are 0.183 Å^3^ and 0.0517 Å^3^. Further, the polarizability of the oxygen from the coordinated ethanol is 4.59 Å^3^, and of the nitrogen atom of 4,4-BPY the value is 1.89 Å^3^, both being intermediary values. This grouping of polarizabilities is in line with what had been the practice until now, and implemented in version 1.0 of LUMPAC^31^. Note that this same grouping also naturally occurs in the RM1 model for Eu(III) for the electronic properties of this same complex (see Table 2). While before, and also in LUMPAC, that had to be done by hand, here the groupings naturally emerge from the quantum chemical calculations.

Table 3 presents the *Q*, *D*, and *C* parameters for similar fittings for all 13 complexes considered, with the electronic densities and superdelocalizabilities computed by single point (using the 1SCF keyword of MOPAC) Sparkle/AM1, whereas Table 4 shows corresponding results computed by single point RM1 model for Eu(III).

Noticeably, a general trend is followed in both Tables 3 and 4 for the quantities *Q*, *D*, and *C*, with all fittings having resulted being acceptably good, except for complex QAMLIB where the errors in both 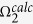 and 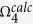 are larger. The same happens when we examine the fittings for the RM1 model for Eu(III), also at the crystallographic geometries.

For both models, the error in 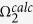 for complex QAMLEX is mildly acceptable, but the error in 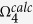 is not. However, the impact of 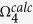 in A_rad_ is much smaller, which makes this situation slightly less important. Assuming that the crystallographic geometries are correct, these inaccurate fittings may have resulted from the Sparkle/RM1 electronic properties, from incorrect experimental values of 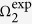 and 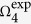, or from an intrinsic inadequacy of the whole model. That is open to investigation. Nevertheless, for all other complexes the obtained fittings were very good.

We then decided to verify what would happen if the crystallographic geometries were not available. To simulate this situation, we then optimized the geometry of the complexes by both RM1 model for Eu(III) and Sparkle/RM1, and carried out the fittings. Results are presented in Tables 5 and 6.

In Table 5, five of the geometries clearly seemed to have been incorrectly predicted and the model could not properly carry out the fitting. For all these five cases (complexes of CSD codes GIPCAK, QAMLIB, VENLEH, VENLIL, and YETTUN) the ratio *D/C* was found to be close to zero after the fitting attempt. One other borderline case, where the ratio *D/C* was found to be 1.00 au^−1^, was OTOYEC. Despite 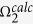 being close to 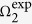, the same could not be said of 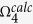. Hence the ratio *D/C* seems to truly function as a compass in pointing to the acceptability of the fit. We are simply presenting these fits in Table 5 to illustrate the cases when we needed to reject the fits as devoid of physical meaning. The cells in the lines corresponding to these fits have been painted gray, so as not to be confused with the other acceptable ones.

The fact that the fits are acceptable when we use crystallographic geometries, and sometimes are not when we use Sparkle Model geometries, suggests that the adjustments seem to fail when the predicted geometries are not sufficiently accurate. This indicates that the choice of the semiempirical model to carry out the geometry optimization is a crucial step in this process. Since the RM1 model for Eu(III) is a more accurate model in terms of obtaining geometries, we expect that the fits will be more successful in this case. And that is corroborated by the results in Table 6, which shows fits from both geometric and electronic properties from RM1 model for Eu(III) calculations. This time, only one fit needed to be rejected due to the fact that the ratio *D/C* was close to zero: the fit for complex DEVHOC.

Accordingly, results presented in Tables 5 and 6 do reinforce the fact that excellent geometries are an important requirement for the fitting to be successful.

The robustness of the fitting can be strengthened by the relative similarity and stability of parameters *Q*, *D*, and *C* across all tables. Indeed, for example, for complex RATKUU, the values of *Q* in Tables 3, 4, 5, 6 are: 0.0546 au^−1^, 0.0550 au^−1^, 0.0275 au^−1^, and 0.193 au^−1^. The corresponding values of *D* are: 60.2 au^−1^.Å^3^, 46.7 au^−1^.Å^3^, 47.6 au^−1^.Å^3^, and 47.5 au^−1^.Å^3^. And the corresponding values for *C* are: 27.6 Å^3^, 20.0 Å^3^, 24.4 Å^3^, and 22.1 Å^3^.

In all cases studied, with either crystallographic or theoretically optimized geometries, only a single minimum could be found in the fit given the constraints imposed on the problem: *Q* > 0, *D* > 0, *C* > −*SE*_*i*_·*D*, and *D* ≈ 2*C*, a result consistent with the uniqueness of the fits being introduced here. Such uniqueness makes possible eventual future interpretations of the meanings of the quantities *Q*, *D*, and *C*.

In the Supplementary Information, we present tables with results for both single point calculations at the experimental geometries, as well as for fully optimized geometries, for all the other Sparkle Models: Sparkle/AM1, Sparkle/PM3, Sparkle/PM6, and Sparkle/PM7.

We now have enough data to test the hypothesis that a geometry, closer to the crystallographic one, will tend to produce more acceptable fittings as measured by the binary outcome acceptance attribute for the adjustment represented by *D/C*, where *D/C* > 1, for acceptable fittings and *D/C *≤ 1 for unacceptable ones. For all complexes, we measured the difference between the theoretically predicted coordination polyhedron (Eu(III) included) and the crystallographic one, by means of their minimized root-mean-square deviation, RMSD, corrected for the number of atoms. The minimization was performed on the polyhedra by translation and rotation, via the Kabsch algorithm^51^ employing a freely available Python script (http://github.com/charnley/rmsd).

Table 7 shows all RMSD values for all complexes considered, computed between the crystallographic coordination polyhedra and the theoretically predicted ones, for all semiempirical models taken into consideration. When the theoretical intensity parameter adjustment resulted unacceptable, we painted the respective cell gray. As a result, we have 27 gray cells and 51 other ones. The mean RMSD of the gray cells is 0.525 Å and the mean RMSD of the 51 other cells is 0.367 Å. This indicates that, indeed, when the error in the geometry of the theoretical coordination polyhedron is larger, the theoretical intensity parameter adjustment tends to result unacceptable. We now turn to quantify the statistical significance of this statement by verifying whether the mean RMSD of the gray cells, 0.525 Å, is truly larger than the mean RMSD of the other cells, 0.367 Å. The t-statistic for both sets of data is 3.089, which, for 26 degrees of freedom, does indicate that the mean for the gray cells is indeed larger than the mean for the regular cells within a 99.8% confidence level.

This result does reinforce the fact that the choice of the semiempirical model to carry out the geometry optimization is truly a crucial step in this process, because one needs an accurate geometry for an acceptable adjustment of the theoretical intensity parameters.

Moreover, if one does not possess the crystallographic geometry and computes the geometries of the complex by two theoretical methods, and one of them leads to an acceptable adjustment, and the other does not, it is likely that the geometry of the one, which yielded the acceptable adjustment, will be closer to the crystallographic geometry.

## Conclusions

In this article, we advanced a procedure for fitting, in a unique manner, the theoretical intensity parameters 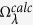 to reproduce the experimentally obtained 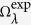 from either crystallographic geometries, or from geometries obtained from Sparkle Model or RM1 calculations on complexes. Thus, we now have a procedure, which is seemingly a robust one, and that leads to a unique set of *g* and α necessary for the prediction of the intensity parameters. The relative stability of the *Q*, *D*, and *C* parameters for the same complex when the semiempirical method employed is varied, as can be seen from Tables 3, 4, 5, 6, as well as from the tables in the Supplementary Material, further strengthens the uniqueness of the adjustment being advanced in this article. In addition, in the absence of crystallographic geometries, these can be obtained from either one of the Sparkle Models^24^,^25^,^26^,^27^,^28^, or from the more accurate RM1 model for lanthanides^30^,^52^.

The model contains a built in quality control index, the ratio *D/C*, which suggests that, in general, something seems not to be correct with the geometry vis a vis the intensity parameters when the value of this ratio is close to zero. Besides, for the adjustment to occur in a perfect manner, a requirement of the whole luminescence model seems to be that the geometry and intensity parameter values must be consistent with each other. In the absence of crystallographic geometries, one can try to optimize the complex with different Sparkle Models or with the RM1 model for lanthanides until a good fit is obtained. That is because each Sparkle Model underlying semiempirical method, AM1, PM3, PM6, PM7, or RM1, treats every type of ligand differently, and one method may be better for some ligand characteristics than others.

As an additional evidence of consistency of our model, we showed that semiempirical methods that lead to an acceptable theoretical intensity parameter adjustment, also tend to produce more accurate geometries, likely closer to the true crystallographic one.

The uniqueness of the adjustment has a number of very good consequences for luminescence research, since, as mentioned before, a unique set of parameters *Q*, *D*, and *C* will lead to a single predicted 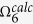 value. Further, the contribution to the intensity parameters from dynamic coupling, (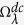), which depends on α_i_, and from electric dipole (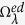), which depends on *g*_i_, will also be unique for any given geometry and any two given values of 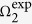 and 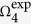. Furthermore, 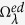, which is used to predict the energy transfer rates via the multipolar mechanism, will be also unique.

## Methods

All Sparkle calculations were carried out using MOPAC2012^29^, and all RM1 model for europium calculations were carried out by a modified version of the same software. Calculations were done either at the crystallographic geometry, or by fully optimizing the geometry at the particular level of theory, when great care was taken to ensure that no imaginary vibrational frequencies were present. A modified version of LUMPAC was then coded to implement the new methodology being advanced in this article, and will be made available as a new version at http://www.lumpac.pro.br. This modified version was used to obtain the results presented in Tables 2, 3, 4, 5, 6, 7 and Tables S1 to S16 of the Supplementary Information.

## Additional Information

**How to cite this article**: Dutra, J. D. L. *et al.* Europium Luminescence: Electronic Densities and Superdelocalizabilities for a Unique Adjustment of Theoretical Intensity Parameters. *Sci. Rep.* **5**, 13695; doi: 10.1038/srep13695 (2015).

## Supplementary Material

Supplementary Information

## Figures and Tables

**Table 1:** Europium complexes with crystallographic structures available from the Cambridge Structural Database, CSD, and that have published values of 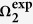 and 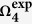.

CSD Code	Complex^a^	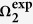 (10^−20^ cm^2^)	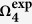 (10^−20^ cm^2^)	Reference
854429^b^	(EMIm)_2_[Eu(PIC)_4_(H_2_O)_2_]PIC	16.7	7.7	53
DEVHOC	Eu(ISOVIND)_3_(H_2_O)(EtOH)	40.9	17.5	54
EWOCOJ	Eu(FOD)_3_(PHEN)	19	2.6	55
GIPCAK	Eu(BTFA)_3_(4,4-BPY)(EtOH)	28.8	6.7	56
LOLXAN	Eu_2_(CYN)_6_(BPY)_2_	7.17	8.96	57
OTOYEC	Eu(BMDM)_3_(TPPO)	37.2	3.1	58
QAMLEX	Eu(TFNB)_3_(PHEN)	46.3	7.8	59
QAMLIB	Eu(PFNP)_3_(PHEN)	49	8.2	59
RATKUU	Eu(DMB)_3_(DMA)	51	6.7	54
VENLEH	(BEIm)_2_[Eu(PIC)_5_]	12	10.3	38
VENLIL	(BBIm)_2_[Eu(PIC)_5_]	9.6	9.2	38
YETTOH	Eu(PBI)_3_(PHEN)	15.66	1.53	60
YETTUN	Eu(PBI)_3_(H_2_O)(EtOH)	16.47	14.29	60

^a^Ligands are identified by the usual abbreviations. EMIm = 1-ethyl-3-methylimidazolium; PIC = picrate; ISOVIND = 2-isovaleryl-1,3-indandionate; EtOH = ethanol; FOD = 6,6,7,7,8,8,8-heptafluoro-2,2-dimethyl-3,5-octadionate; PHEN = 1,10-phenanthroline; BTFA = 4,4,4-trifluoro-1-phenyl-2,4-butanedione; 4,4-BPY = 4,4′-bipyridine; CIN = hydrocinnamate; BPY = 2,2′-bipyridine; BMDM = methoxy-dibenzoyl-methane; TPPO = triphenylphosphine oxide; TFNB = 4,4,4-trifluoro-1-(2-naphthyl)butane-1,3-dione; PFNP = 4,4,5,5,5-pentafluoro-1-(2-naphthyl)pentane-1,3- dione; DMB = dimethylbenzamide; DMA = dimethylacetamide; BEMIm = 1-butyl-3-ethylimidazolium; BBIm = 1-butyl-3-methylimidazolium; PBI = 3-phenyl-4-benzoyl-5-isoxazolonate.

^b^Cambridge Crystallographic Data Centre deposited CSD entry.

**Table 2:** Sparkle/RM1 and RM1 model for Eu(III) ZDO electronic densities *q* and electrophilic superdelocalizabilities for each atom directly coordinated to europium(III), in complex Eu(BTFA)_3_(4,4-BPY)(EtOH), CSD code GIPCAK, together with corresponding charge factors *g* and polarizabilities α from the fitting.

	Sparkle/RM1				RM1 model for Eu(III)			
Ligand Atom	*Q* = 0.0537 au^−1^				*Q* = 0.0927 au^−1^			
	*D* = 25.5 au^−1^·Å^3^				*D* = 31.4 au^−1^·Å^3^			
	*C* = 12.0 Å^3^				*C* = 12.9 Å^3^			
	*D/C* = 2.13 au^−1^				*D/C* = 2.43 au^−1^			
	*q* (au)	*SE* (au)	*g*	α (Å^3^)	*q* (au)	*SE* (au)	*g*	α (Å^3^)
N2 (4,4-BPY)	5.50	−0.395	0.296	1.89	5.22	−0.344	0.484	2.15
O3 (BTFA1)	6.67	−0.461	0.358	0.183	6.32	−0.399	0.586	0.413
O4 (BTFA1)	6.73	−0.466	0.362	0.0517	6.35	−0.412	0.588	0.0081
O5 (BTFA2)	6.76	−0.418	0.363	1.30	6.37	−0.351	0.591	1.94
O6 (BTFA2)	6.67	−0.420	0.358	1.23	6.35	−0.380	0.589	1.01
O7 (BTFA3)	6.71	−0.203	0.361	6.78	6.32	−0.193	0.587	6.87
O8 (BTFA3)	6.73	−0.196	0.362	6.97	6.36	−0.189	0.590	7.03
O9 (EtOH)	6.53	−0.289	0.351	4.59	6.28	−0.270	0.582	4.48

Calculated values for the intensity parameters agreed with the experimentally determined ones in both cases, where 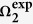 = 28.8 × 10^−20^ cm^2^ and 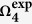
** = **6.7 × 10^−20^ cm^2^.

**Table 3:** Fitted *Q*, *D*, and *C* values for all complexes studied, with electronic densities and electrophilic superdelocalizabilities computed by single point (1SCF) Sparkle/RM1 at the crystallographic geometries, together with calculated and experimental Ω_*λ*_values†.

CSD code	*Q*	*D*	*C*	*D/C*	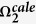	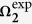	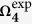	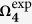	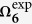
854429^a^	0.0454	19.9	11.1	1.79	16.7	16.7	7.70	7.7	0.149
DEVHOC	0.0338	38.6	19.7	1.96	40.9	40.9	17.5	17.5	0.338
EWOCOJ	0.260	33.3	16.7	1.99	18.7	19	3.70	2.6	1.25
GIPCAK	0.0537	25.5	12.0	2.13	28.8	28.8	6.70	6.7	0.246
LOLXAN	0.0416	16.4	10.9	1.50	7.17	7.17	8.97	8.96	0.0917
OTOYEC	0.271	58.0	29.2	1.99	37.1	37.2	4.33	3.1	1.18
QAMLEX	0.296	49.0	24.2	2.03	45.4	46.3	10.9	7.8	2.04
QAMLIB	0.297	52.6	26.7	1.97	38.7	49	7.57	8.2	1.88
RATKUU	0.0546	60.2	27.6	2.18	51.0	51	6.71	6.7	0.206
VENLEH	0.0372	17.6	10.3	1.71	12.0	12	10.3	10.3	0.153
VENLIL	0.0177	22.2	10.7	2.08	9.60	9.6	9.22	9.2	0.126
YETTOH	0.277	39.8	18.6	2.13	15.3	15.66	3.04	1.53	1.37
YETTUN	0.0729	29.0	14.0	2.06	16.5	16.47	14.3	14.29	0.307

^†^Units are: *Q* (au^−1^); *D* (au^−1^·Å^3^); *C* (Å^3^); *D/C* (au^−1^); Ω_*λ*_(10^−20^ cm^2^).

^a^Cambridge Crystallographic Data Centre deposited CSD entry.

**Table 4:** Fitted *Q*, *D*, and *C* values for all complexes studied with electronic densities and electrophilic superdelocalizabilities computed by single point (1SCF) RM1 model for Eu(III) at the crystallographic geometries, together with calculated and experimental Ω_*λ*_ values†.

CSD code	*Q*	*D*	*C*	*D/C*	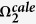	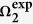	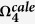	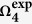	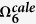
854429^a^	0.0442	23.3	11.6	2.00	16.7	16.7	7.70	7.7	0.151
DEVHOC	0.0292	39.8	18.5	2.15	40.9	40.9	17.5	17.5	0.227
EWOCOJ	0.315	36.6	16.7	2.19	18.0	19	5.15	2.6	1.62
GIPCAK	0.0927	31.4	12.9	2.43	28.8	28.8	6.75	6.7	0.277
LOLXAN	0.0435	25.2	13.0	1.93	7.17	7.17	8.96	8.96	0.0979
OTOYEC	0.292	68.4	30.8	2.22	36.3	37.2	6.78	3.1	1.14
QAMLEX	0.315	54.5	24.3	2.24	44.3	46.3	12.7	7.8	1.99
QAMLIB	0.315	59.8	28.0	2.14	40.9	49	12.8	8.2	1.80
RATKUU	0.0550	46.7	20.0	2.34	51.0	51	6.68	6.7	0.173
VENLEH	0.0402	20.1	10.6	1.90	12.0	12	10.3	10.3	0.158
VENLIL	0.0239	24.4	10.7	2.27	9.61	9.6	9.20	9.2	0.121
YETTOH	0.314	39.0	16.9	2.30	15.0	15.66	3.46	1.53	1.52
YETTUN	0.0855	32.6	14.3	2.28	16.5	16.47	14.3	14.29	0.283

^†^Units are: *Q* (au^−1^); *D* (au^−1^.Å^3^); *C* (Å^3^); *D/C* (au^−1^); Ω_*λ*_ (10^−20^cm^2^).

^a^Cambridge Crystallographic Data Centre deposited CSD entry.

**Table 5:**
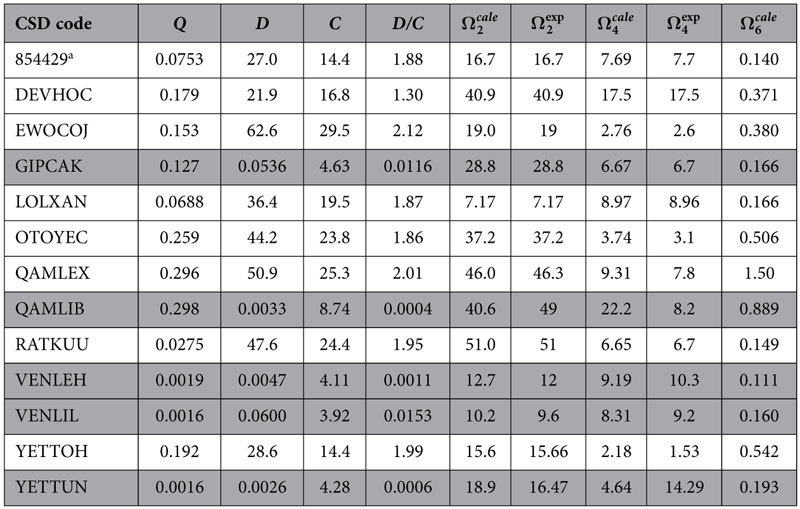
Fitted *Q*, *D*, and *C* values for all complexes studied, with electronic densities and electrophilic superdelocalizabilities computed by Sparkle/RM1 at Sparkle/RM1 fully optimized geometries, together with calculated and experimental Ω_*λ*_ values^†^.

The cells corresponding to geometries which led to unacceptable theoretical intensity parameters are painted gray[Fn t5-fn1].

^†^Units are: *Q* (au^−1^); *D* (au^−1^·Å^3^); *C* (Å^3^); *D/C* (au^−1^);Ω_*λ*_ (10^−20^cm^2^).

^a^Cambridge Crystallographic Data Centre deposited CSD entry.

**Table 6:**
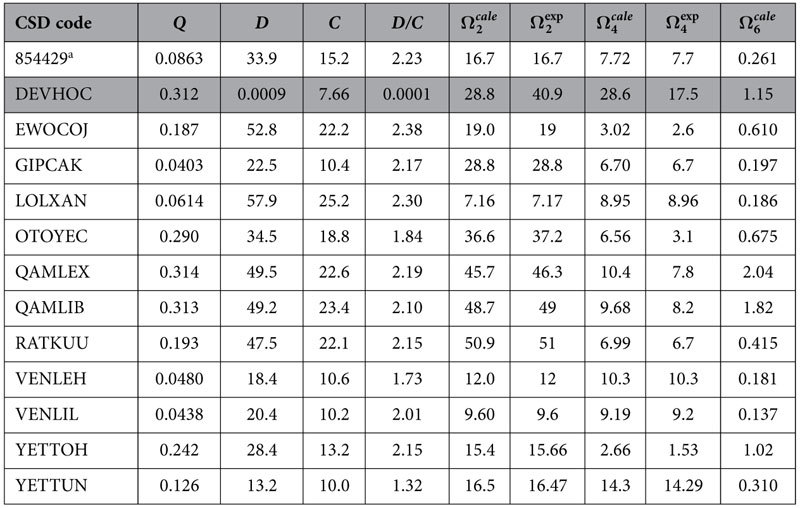
Fitted *Q*, *D*, and *C* values for all complexes studied. with electronic densities and electrophilic superdelocalizabilities computed by RM1 for Eu(III) at RM1 for Eu(III) fully optimized geometries, together with calculated and experimental Ω_*λ*_ values^†^.

The cells corresponding to geometries which led to unacceptable theoretical intensity parameters are painted gray.

^†^Units are: *Q* (au^−1^); *D* (au^−1^.Å^3^); *C* (Å^3^); *D/C* (au^−1^); Ω_*λ*_ (10^−20^cm^2^).

^a^Cambridge Crystallographic Data Centre deposited CSD entry.

**Table 7:**
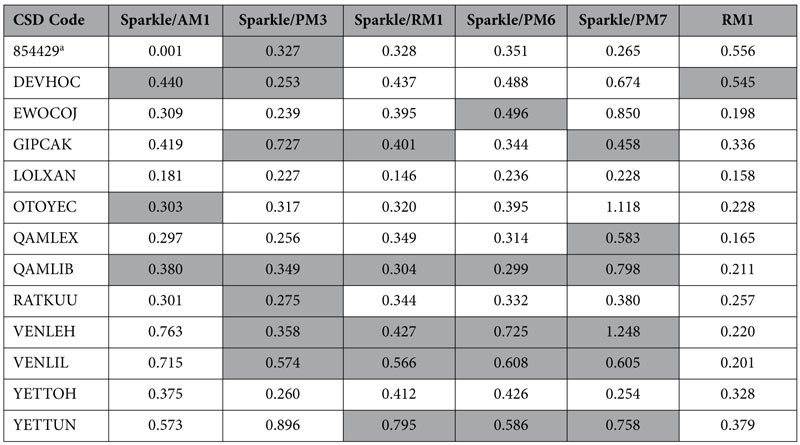
RMSD values for all complexes considered between the crystallographic coordination polyhedra geometries and the fully optimized theoretical ones for all semiempirical methods available for lanthanide complexes^†^.

The cells corresponding to geometries which led to unacceptable theoretical intensity parameters (see Tables 5, 6 of this article and S13 to S16 of the Supplementary Material) are painted gray.

^†^Units are Å.

^a^Cambridge Crystallographic Data Centre deposited CSD entry.

## References

[b1] Bethe H. A. Splitting of Terms in Crystals. Ann. Physik 3, 133–206 (1929).

[b2] Vleck J. H. V. The Puzzle of Rare-earth Spectra in Solids. J. Phys. Chem. 41, 67–80 (1937).

[b3] Broer L. J. F., Gorter C. J. & Hoogschagen J. On the intensities and the multipole character in the spectra of the rare earth ions. Physica 11, 231–250 (1945).

[b4] Judd B. R. Optical Absorption Intensities of Rare-Earth Ions. Phys. Rev. 127, 750–& (1962).

[b5] Ofelt G. S. Intensities of Crystal Spectra of Rare-Earth Ions. J. Chem. Phys. 37, 511–& (1962).

[b6] Jørgensen C. K., Pappalardo R. & Schmidtke H. H. Do the “Ligand Field” Parameters in Lanthanides Represent Weak Covalent Bonding? J. Chem. Phys. 39, 1422–1430 (1963).

[b7] Burns G. & Axe J. D. Covalent Bonding Effects in Rare Earth Crystal Fields, in Optical properties of ions in crystals. (eds. Crosswhite H. M. & Moos H. W. ) 53–71 (Interscience Publishers, 1967).

[b8] Newman D. J. Theory of lanthanide crystal fields. Adv. Phys. 20, 197–& (1971).

[b9] Malta O. L. A Simple Overlap Model in Lanthanide Crystal-Field Theory. Chem. Phys. Lett. 87, 27–29 (1982).

[b10] Jørgensen C. K. & Judd B. R. Hypersensitive pseudoquadrupole transitions in lanthanides. Mol. Phys. 8, 281–290 (1964).

[b11] Malta O. L., Batista H. J. & Carlos L. D. Overlap polarizability of a chemical bond: a scale of covalency and application to lanthanide compounds. Chem. Phys. 282, 21–30 (2002).

[b12] de Andrade A. V. M., da Costa N. B., Simas A. M. & de Sa G. F. Sparkle Model for the Quantum-Chemical AM1 Calculation of Europium Complexes. Chem. Phys. Lett. 227, 349–353 (1994).

[b13] de Andrade A. V. M., da Costa N. B., Simas A. M. & de Sa G. F. Sparkle Model for the Quantum-Chemical AM1 Calculation of Europium Complexes of Coordination-Number-9. J. Alloy. Compd. 225, 55–59 (1995).

[b14] de Andrade A. V. M., Longo R. L., Simas A. M. & de Sa G. F. Theoretical model for the prediction of electronic spectra of lanthanide complexes. Faraday Trans. 92, 1835–1839 (1996).

[b15] Rocha G. B., Freire R. O., da Costa N. B., de Sá G. F. & Simas A. M. Sparkle Model for AM1 Calculation of Lanthanide Complexes: Improved Parameters for Europium. Inorg. Chem. 43, 2346–2354 (2004). 15046511 10.1021/ic034882p

[b16] Faustino W. M. *et al.* Design of ligands to obtain lanthanide ion complexes displaying high quantum efficiencies of luminescence using the sparkle model. J. Mol. Struc. Theochem 527, 245–251 (2000).

[b17] Freire R. O., Rocha G. B., Albuquerque R. Q. & Simas A. M. Efficacy of the semiempirical sparkle model as compared to ECP ab-initio calculations for the prediction of ligand field parameters of europium(III) complexes. J. Lumin. 111, 81–87 (2005).

[b18] Dewar M. J. S., Zoebisch E. G., Healy E. F. & Stewart J. J. P. The Development and Use of Quantum Mechanical Molecular models. 76. AM1 - A New General Purpose Quantum Mechanical Molecular Model. J. Am. Chem. Soc. 107, 3902–3909 (1985).

[b19] Stewart J. J. P. Optimization of Parameters for Semiempirical Methods. 2. Applications. J. Comput. Chem. 10, 221–264 (1989).

[b20] Stewart J. J. P. Optimization of Parameters for Semiempirical Methods. 1. Method. J. Comput. Chem. 10, 209–220 (1989).

[b21] Stewart J. J. P. Optimization of parameters for semiempirical methods V: Modification of NDDO approximations and application to 70 elements. J. Mol. Model. 13, 1173–1213 (2007). 17828561 10.1007/s00894-007-0233-4 PMC2039871

[b22] Stewart J. J. P. Optimization of parameters for semiempirical methods VI: more modifications to the NDDO approximations and re-optimization of parameters. J. Mol. Model. 19, 1–32 (2013). 23187683 10.1007/s00894-012-1667-x PMC3536963

[b23] Rocha G. B., Freire R. O., Simas A. M. & Stewart J. J. P. RM1: A reparameterization of AM1 for H, C, N, O, P, S, F, Cl, Br, and I. J. Comput. Chem. 27, 1101–1111 (2006). 16691568 10.1002/jcc.20425

[b24] Freire R. O., Rocha G. B. & Simas A. M. Sparkle model for the calculation of lanthanide complexes: AM1 parameters for Eu(III), Gd(III), and Tb(III). Inorg. Chem. 44, 3299–3310 (2005). 15847440 10.1021/ic048530+

[b25] Freire R. O., Rocha G. B. & Simas A. M. Sparkle/PM3 for the Modeling of Europium(III), Gadolinium(III), and Terbium(III) Complexes. J. Brazil Chem. Soc. 20, 1638–1645 (2009).

[b26] Freire R. O. & Simas A. M. Sparkle/PM6 Parameters for all Lanthanide Trications from La(III) to Lu(III). J. Chem. Theory Comput. 6, 2019–2023 (2010). 10.1021/ct100192c 26615930

[b27] Dutra J. D. L. *et al.* Sparkle/PM7 Lanthanide Parameters for the Modeling of Complexes and Materials. J. Chem. Theory Comput. 9, 3333–3341 (2013). 24163641 10.1021/ct301012h PMC3806451

[b28] Filho M. A. M., Dutra J. D. L., Rocha G. B., Freire R. O. & Simas A. M. Sparkle/RM1 parameters for the semiempirical quantum chemical calculation of lanthanide complexes. RSC Adv. 3, 16747–16755 (2013).

[b29] MOPAC2012, Stewart J. J. P. Stewart Computational Chemistry, Colorado Springs, CO, USA, 2012 (http://OpenMOPAC.net).

[b30] Filho M. A. *et al.* RM1 Model for the Prediction of Geometries of Complexes of the Trications of Eu, Gd, and Tb. J. Chem. Theory Comput. 10, 3031–3037 (2014). 10.1021/ct400909w 26588274

[b31] Dutra J. D. L., Bispo T. D. & Freire R. O. LUMPAC lanthanide luminescence software: Efficient and user friendly. J. Comput. Chem. 35, 772–775 (2014). 24532191 10.1002/jcc.23542

[b32] Dutra J. D. L. & Freire R. O. Theoretical tools for the calculation of the photoluminescent properties of europium systems—A case study. J. Photoch. Photobio. A 256, 29–35 (2013).

[b33] de Sa G. F. *et al.* Spectroscopic properties and design of highly luminescent lanthanide coordination complexes. Coord. Chem. Rev. 196, 165–195 (2000).

[b34] Malta O. L. *et al.* Experimental and theoretical study of ligand field, 4f-4f intensities and emission quantum yield in the compound Eu(bpyO(2))(4)(ClO4)(3). J. Alloy. Compd. 323, 654–660 (2001).

[b35] da Costa N. B., Freire R. O., dos Santos M. A. C. & Mesquita M. E. Sparkle model and intensity parameters of the Eu(3-amino-2-carboxypyridine-N-oxide)_3_.3H_2_O complex. J. Mol. Struc. Theochem 545, 131–135 (2001).

[b36] de Mesquita M. E. *et al.* Synthesis, sparkle model, intensity parameters and spectroscopic studies of the new Eu(fod)_3_phen-NO complex. J. Solid State Chem. 171, 183–188 (2003).

[b37] Biju S., Reddy M. L. P. & Freire R. O. 3-phenyl-4-aroyl-5-isoxazolonate complexes of Tb^3+^ as promising light-conversion molecular devices. Inorg. Chem. Commun. 10, 393–396 (2007). 10.1021/ic051781d 16499381

[b38] Borges A. S. *et al.* Synthesis and Characterization of the Europium(III) Pentakis(picrate) Complexes with Imidazolium Countercations: Structural and Photoluminescence Study. Inorg. Chem. 51, 12867–12878 (2012). 23151323 10.1021/ic301776n

[b39] Rodrigues C. V. *et al.* Unusual photoluminescence properties of the 3D mixed-lanthanide-organic frameworks induced by dimeric structures: a theoretical and experimental approach. Phys. Chem. Chem. Phys. 16, 14858–14866 (2014). 24924492 10.1039/c4cp00405a

[b40] Simas A. M. Aplicação de Índices Derivados Para o Método CNDO aos Estudos de Reatividade Química e Atividade Biológica de Drogas, in Instituto de Química, Master of Sciences Dissertation (Universidade Estadual de Campinas, Campinas, Brazil, 1977).

[b41] Malta O. L. Theoretical Crystal-Field Parameters for the YOCl:Eu^3+^ System—A Simple Overlap Model. Chem. Phys. Lett. 88, 353–356 (1982).

[b42] Fukui K., Yonezawa T., Nagata C. & Shingu H. Molecular Orbital Theory of Orientation in Aromatic, Heteroaromatic, and Other Conjugated Molecules. J. Chem. Phys. 22, 1433–1442 (1954).

[b43] Lewis D. F. V. Molecular orbital calculations on solvents and other small molecules: Correlation between electronic and molecular properties ν, αMOL, π*, and β. J. Comput. Chem. 8, 1084–1089 (1987).

[b44] Brown R. E. & Simas A. M. On the Applicability of CNDO Indexes for the Prediction of Chemical-Reactivity. Theor. Chim. Acta 62, 1–16 (1982).

[b45] Schüürmann G. QSAR analysis of the acute fish toxicity of organic phosphorothionates using theoretically derived molecular descriptors. Environ. Toxicol. Chem. 9, 417–428 (1990).

[b46] Schüürmann G. Ecotoxicology and structure-activity studies of organophosphorus compounds, in Rational Approaches to Structure, Activity, and Ecotoxicology of Agrochemicals. Edn. 1 edition (eds. Fujita T. & Draber W. ) 485–541 (CRC Press, 1992).

[b47] Tsallis C. & Stariolo D. A. Generalized simulated annealing. Physica A 233, 395–406 (1996).

[b48] Allen F. H. The Cambridge Structural Database: a quarter of a million crystal structures and rising. Acta Crystallogr. B 58, 380–388 (2002). 12037359 10.1107/s0108768102003890

[b49] Allen F. H. & Motherwell W. D. S. Applications of the Cambridge Structural Database in organic chemistry and crystal chemistry. Acta Crystallogr. B 58, 407–422 (2002). 12037362 10.1107/s0108768102004895

[b50] Bruno I. J. *et al.* New software for searching the Cambridge Structural Database and visualizing crystal structures. Acta Crystallogr. B 58, 389–397 (2002). 12037360 10.1107/s0108768102003324

[b51] Kabsch W. A solution for the best rotation to relate two sets of vectors. Acta Crystallogr. A 32, 922–923 (1976).

[b52] Filho M. A. M., Dutra J. D. L., Rocha G. B., Simas A. M. & Freire R. O. Semiempirical Quantum Chemistry Model for the Lanthanides: RM1 (Recife Model 1) Parameters for Dysprosium, Holmium and Erbium. Plos One 9 (2014). 10.1371/journal.pone.0086376 PMC3908927 24497945

[b53] Borges A. S. *et al.* Synthesis, crystal structure and luminescence properties of the Ln(III)-picrate complexes with 1-ethyl-3-methylimidazolium as countercations. Spectrochim. Acta A 117, 718–727 (2014). 10.1016/j.saa.2013.08.039 24140743

[b54] Teotonio E. E. S. *et al.* Synthesis and luminescent properties of Eu^3+^-complexes with 2-acyl-1,3-indandionates (ACIND) and TPPO ligands: The first X-ray structure of Eu-ACIND complex. Polyhedron 25, 3488–3494 (2006).

[b55] dos Santos E. R. *et al.* Theoretical and Experimental Spectroscopic Approach of Fluorinated Ln^3+^ -beta-Diketonate Complexes. J. Phys. Chem. A 114, 7928–7936 (2010). 20617802 10.1021/jp104038r

[b56] Lima P. P. *et al.* Spectroscopic study of a UV-photostable organic-inorganic hybrids incorporating an Eu^3+^ beta-diketonate complex. Chemphyschem 7, 735–746 (2006). 16514701 10.1002/cphc.200500588

[b57] Marques L. F. *et al.* Theoretical and Experimental Spectroscopic Studies of the First Highly Luminescent Binuclear Hydrocinnamate of Eu(III), Tb(III) and Gd(III) with Bidentate 2,2′-Bipyridine Ligand. J. Lumin. 148, 307–316 (2014).

[b58] Monteiro J. H. S. K., Adati R. D., Davolos M. R., Vicenti J. R. M. & Burrow R. A. Correlation between structural data and spectroscopic studies of a new beta-diketonate complex with trivalent europium and gadolinium. New J .Chem. 35, 1234–1241 (2011).

[b59] Yu J. B., Deng R. P., Sun L. N., Li Z. F. & Zhang H. J. Photophysical properties of a series of high luminescent europium complexes with fluorinated ligands. J. Lumin. 131, 328–335 (2011).

[b60] Biju S., Raj D. B. A., Reddy M. L. P. & Kariuki B. M. Synthesis, crystal structure, and luminescent properties of novel Eu^3+^ heterocyclic beta-diketonate complexes with bidentate nitrogen donors. Inorg. Chem. 45, 10651–10660 (2006). 17173420 10.1021/ic061425a

