# Supplementary material for: Europium Luminescence: Electronic Densities and Superdelocalizabilities for a Unique Adjustment of Theoretical Intensity Parameters
Source: Sci Rep. 2015 Sep 2;5:13695. doi: 10.1038/srep13695 (PMC4557129; doi:10.1038/srep13695)
Supplement: Supplementary Information [file srep13695-s1.pdf]

Europium Luminescence: Electronic Densities and Superdelocalizabilities for a Unique Adjustment of Theoretical Intensity Parameters

Supplementary Information

*José Diogo L. Dutra[a,b], Nathália B. Lima[b], Ricardo O. Freire[a], and Alfredo M. Simas[b]**

[a] Pople Computational Chemistry Laboratory, Departamento de Química, CCET, UFS, 49100-000 - Aracaju, SE, Brazil.

[b] Departamento de Química Fundamental, CCEN, UFPE, 50590-470 – Recife, PE, Brazil.

* Corresponding author. Tel. +55 81 2126-8434; Fax: +55 81 2126-8442.

E-mail: simas@ufpe.br

Summary

Europium luminescence charge factors g_i and polarizabilities α_i 7

1. Experimental Radiative Decay Rates from Emission Spectra 7

2. Theoretical Radiative Decay Rates 9

3. Perturbation theory formalism on the semiempirical wavefunctions 12

4. g_i charges from the semiempirical models 17

5. α_i polarizabilities from the semiempirical models 18

References.. 23

Additional Figures and Tables 24

List of Figures

Figure S1. Perspective view of the crystallographic geometry of complex **$(EMIm)_2[Eu(PIC)_4(H_2O)_2]PIC$**, CSD deposited entry 854429.................................. 24

Figure S2. Perspective view of the crystallographic geometry of complex **$Eu(ISOVIND)_3(H_2O)(EtOH)$**, CSD code **DEVHOC**. 25

Figure S3. Perspective view of the crystallographic geometry of complex **$Eu(FOD)_3(PHEN)$**, CSD code **EWOCOJ**. 26

Figure S4. Perspective view of the crystallographic geometry of complex **$Eu_2(CYN)_6(BPY)_2$**, CSD code **LOLXAN**. 27

Figure S5. Perspective view of the crystallographic geometry of complex **$Eu(BMDM)_3(TPPO)$**, CSD code **OTOYEC**. 28

Figure S6. Perspective view of the crystallographic geometry of complex **$Eu(PFNP)_3(PHEN)$**, CSD code **QAMLEX**. 29

Figure S7. Perspective view of the crystallographic geometry of complex **$Eu(TFNB)_3(PHEN)$**, CSD code **QAMLIB**. 30

Figure S8. Perspective view of the crystallographic geometry of complex **$Eu(DMB)_3(DMA)$**, CSD code **RATKUU**. 31

Figure S9. Perspective view of the crystallographic geometry of complex **$(BEIm)_2[Eu(PIC)_5]$**, CSD code **VENLEH**. 32

Figure S10. Perspective view of the crystallographic geometry of complex **$(BBIm)_2[Eu(PIC)_5]$**, CSD code **VENLIL**. 33

Figure S11. Perspective view of the crystallographic geometry of complex **$Eu(PBI)_3(PHEN)$**, CSD code **YETTOH**. 34

Figure S12. Perspective view of the crystallographic geometry of complex **$Eu(PBI)_3(H_2O)(EtOH)$**, CSD code **YETTUN**. 35

List of Tables

Table S1. Sparkle/RM1 and RM1 model for Eu(III) ZDO electronic densities *q* and electrophilic superdelocalizabilities *SE* for each atom directly coordinated to europium(III), in complex **$(EMIm)_2[Eu(PIC)_4(H_2O)_2]PIC$**, CSD deposited entry 854429, at the crystallographic geometry, together with corresponding charge factors *g* and polarizabilities α from the fitting. 24

Table S2. Sparkle/RM1 and RM1 model for Eu(III) ZDO electronic densities *q* and electrophilic superdelocalizabilities *SE* for each atom directly coordinated to europium(III), in complex **$Eu(ISOVIND)_3(H_2O)(EtOH)$**, CSD code **DEVHOC**, at the crystallographic geometry, together with corresponding charge factors *g* and polarizabilities α from the fitting. 25

Table S3. Sparkle/RM1 and RM1 model for Eu(III) ZDO electronic densities *q* and electrophilic superdelocalizabilities *SE* for each atom directly coordinated to europium(III), in complex **$Eu(FOD)_3(PHEN)$**, CSD code **EWOCOJ**, at the crystallographic geometry, together with corresponding charge factors *g* and polarizabilities α from the fitting. 26

Table S4. Sparkle/RM1 and RM1 model for Eu(III) ZDO electronic densities *q* and electrophilic superdelocalizabilities *SE* for each atom directly coordinated to europium(III), in complex **$Eu_2(CYN)_6(BPY)_2$**, CSD code **LOLXAN**, at the crystallographic geometry, together with corresponding charge factors *g* and polarizabilities α from the fitting. 27

Table S5. Sparkle/RM1 and RM1 model for Eu(III) ZDO electronic densities *q* and electrophilic superdelocalizabilities *SE* for each atom directly coordinated to europium(III), in complex **$Eu(BMDM)_3(TPPO)$**, CSD code **OTOYEC**, at the crystallographic geometry, together with corresponding charge factors *g* and polarizabilities α from the fitting. 28

Table S6. Sparkle/RM1 and RM1 model for Eu(III) ZDO electronic densities *q* and electrophilic superdelocalizabilities *SE* for each atom directly coordinated to europium(III), in complex **$Eu(PFNP)_3(PHEN)$**, CSD code **QAMLEX**, at the crystallographic geometry, together with corresponding charge factors *g* and polarizabilities α from the fitting. 29

Table S7. Sparkle/RM1 and RM1 model for Eu(III) ZDO electronic densities *q* and electrophilic superdelocalizabilities *SE* for each atom directly coordinated to europium(III), in complex **$Eu(TFNB)_3(PHEN)$**, CSD code **QAMLIB**, at the crystallographic geometry, together with corresponding charge factors *g* and polarizabilities α from the fitting. 30

Table S8. Sparkle/RM1 and RM1 model for Eu(III) ZDO electronic densities *q* and electrophilic superdelocalizabilities *SE* for each atom directly coordinated to europium(III), in complex **$Eu(DMB)_3(DMA)$**, CSD code **RATKUU**, at the

crystallographic geometry, together with corresponding charge factors *g* and polarizabilities α from the fitting. 31

Table S9. Sparkle/RM1 and RM1 model for Eu(III) ZDO electronic densities *q* and electrophilic superdelocalizabilities *SE* for each atom directly coordinated to europium(III), in complex **(BEIm)$_2$[Eu(PIC)$_5$]**, CSD code **VENLEH**, at the crystallographic geometry, together with corresponding charge factors *g* and polarizabilities α from the fitting. 32

Table S10. Sparkle/RM1 and RM1 model for Eu(III) ZDO electronic densities q and electrophilic superdelocalizabilities *SE* for each atom directly coordinated to europium(III), in complex **(BBIm)$_2$[Eu(PIC)$_5$]**, CSD code **VENLIL**, at the crystallographic geometry, together with corresponding charge factors *g* and polarizabilities α from the fitting. 33

Table S11. Sparkle/RM1 and RM1 model for Eu(III) ZDO electronic densities q and electrophilic superdelocalizabilities *SE* for each atom directly coordinated to europium(III), in complex **Eu(PBI)$_3$(PHEN)**, CSD code **YETTOH**, at the crystallographic geometry, together with corresponding charge factors g and polarizabilities α from the fitting. 34

Table S12. Sparkle/RM1 and RM1 model for Eu(III) ZDO electronic densities q and electrophilic superdelocalizabilities *SE* for each atom directly coordinated to europium(III), in complex **Eu(PBI)$_3$(H$_2$O)(EtOH)**, CSD code **YETTUN**, at the crystallographic geometry, together with corresponding charge factors *g* and polarizabilities α from the fitting. 35

Table S13. Fitted *Q*, *D*, and *C* values for all complexes studied. with electronic densities and electrophilic Superdelocalizabilities computed by **Sparkle/AM1** for Eu(III) at **Sparkle/AM1** fully optimized geometries, together with calculated and experimental Ω_λ values. The cells corresponding to geometries which led to unacceptable theoretical intensity parameters are painted gray.† 36

Table S14. Fitted *Q*, *D*, and *C* values for all complexes studied. with electronic densities and electrophilic Superdelocalizabilities computed by **Sparkle/PM3** for Eu(III) at **Sparkle/PM3** fully optimized geometries, together with calculated and experimental Ω_λ values. The cells corresponding to geometries which led to unacceptable theoretical intensity parameters are painted gray.† 37

Table S15. Fitted *Q*, *D*, and *C* values for all complexes studied. with electronic densities and electrophilic Superdelocalizabilities computed by **Sparkle/PM6** for Eu(III) at **Sparkle/PM6** fully optimized geometries, together with calculated and experimental Ω_λ values. The cells corresponding to geometries which led to unacceptable theoretical intensity parameters are painted gray.† 38

Table S16. Fitted *Q*, *D*, and *C* values for all complexes studied. with electronic densities and electrophilic Superdelocalizabilities computed by **Sparkle/PM7** for Eu(III) at

Sparkle/PM7 fully optimized geometries, together with calculated and experimental Ω_λ values. The cells corresponding to geometries which led to unacceptable theoretical intensity parameters are painted gray.[†] 39

Europium luminescence charge factors g_i and polarizabilities α_i

1. Experimental Radiative Decay Rates from Emission Spectra

When ultraviolet light illuminates a suitable europium complex, its ligands absorb the photons as antennae. Subsequently, the ligands transfer the energy to the trivalent europium ion, which is excited mostly to its $^5D_0$ state. Once in its $^5D_0$ excited state, the trivalent europium ion undergoes both radiative and non-radiative decays in a competitive manner. Radiatively, it goes from its $^5D_0$ state to any of the ^7F_J states, with J ranging from 0 to 6, emitting mainly orangish red light due to the intense $^5D_0$ to $^7F_2$ transition around 612nm ~614nm. The remainder of the energy dissipates non-radiatively. Of course, the more luminescent the complex, the less it will decay via non-radiative channels.

The experimental radiative decay rate, $A_{rad}^{\exp}$, is the sum of the radiative decay rates of each of the possible transitions $^5D_0 \rightarrow {}^7F_J$, with J ranging from 0 to 6, which occur simultaneously:

$$A_{rad}^{\exp} = \sum_{J=0}^{6} A_{rad}^{\exp}[^5D_0 \rightarrow {}^7F_J] \tag{S1}$$

where the transition inside the square brackets serve to further index the quantity before it, in this case $A_{rad}^{\exp}$.

Transitions $^5D_0 \rightarrow {}^7F_J$ with J = 0, 3, and 5, are forbidden by three mechanisms: magnetic dipole, forced electric dipole and dynamic coupling. However, they usually appear – albeit with very low intensities – due to J-mixing effects. We will neglect them in the present work.

The transition $^5D_0 \rightarrow {}^7F_1$ is governed by a magnetic dipole mechanism and does not have electric dipole contributions. It is considered to be insensitive to changes in the chemical environment. It can be determined through the expression $A_{rad}^{\exp}[^5D_0 \rightarrow {}^7F_1] = (0.31\times10^{-11} cm^3 s^{-1}) n^3 \nu[^5D_0 \rightarrow {}^7F_1]^3$, where n is the refractive index of the

medium, and $\nu[^5D_0\rightarrow{}^7F_1]$ is the barycenter of its corresponding spectral line, the weighted mean of the frequencies in cm^{-1} of the $^5D_0\rightarrow{}^7F_1$ transition. From this value, we can now compute the other radiative decay rates, with J from 0 to 6.

$$A_{rad}^{exp}[^5D_0\rightarrow{}^7F_J]=\frac{\nu[^5D_0\rightarrow{}^7F_1]}{\nu[^5D_0\rightarrow{}^7F_J]}\frac{S[^5D_0\rightarrow{}^7F_J]}{S[^5D_0\rightarrow{}^7F_1]}A_{rad}^{exp}[^5D_0\rightarrow{}^7F_1] \qquad \text{(S2)}$$

where $\nu[^5D_0\rightarrow{}^7F_J]$ are the energies of the barycenters of the respective transitions; and $S[^5D_0\rightarrow{}^7F_J]$ are the areas under the spectra corresponding to the respective transitions.

The transition $^5D_0\rightarrow{}^7F_2$ is called the hypersensitive transition[1], which is well described by Judd-Ofelt theory and is remarkably responsive to the immediate chemical environment, namely the coordination polyhedron, around the metal ion, which is inscribed into it.

On the other hand, transitions $^5D_0\rightarrow{}^7F_4$ and $^5D_0\rightarrow{}^7F_6$ are less sensitive to the chemical environment neighboring the lanthanide ion, and are well described by Judd-Ofelt theory.

Finally, the experimental intensity parameters Ω_λ^{exp} can be calculated exclusively for the transitions governed by the forced dipole mechanism, J = 2, 4, 6, from the experimental radiative decay rates $A_{rad}^{exp}[^5D_0\rightarrow{}^7F_J]$ according to:

$$\Omega_{\lambda=J}^{exp}=\frac{3\hbar A_{rad}^{exp}[^5D_0\rightarrow{}^7F_J]}{32e^2\pi^3\chi\nu[^5D_0\rightarrow{}^7F_J]^3\left|\left\langle{}^5D_0\left\|U^{(\lambda)}\right\|{}^7F_J\right\rangle\right|^2} \qquad \text{(S3)}$$

where $\hbar$ is Planck-Dirac constant, e is the fundamental electric charge, χ is the Lorentz local-field correction term given by $\chi=n\left(n^2+2\right)^2/9$, where n is the refractive index of the medium, $\nu[^5D_0\rightarrow{}^7F_J]$ is the frequency of the transition in wavenumbers, and $\left|\left\langle{}^5D_0\left\|U^{(\lambda)}\right\|{}^7F_J\right\rangle\right|^2$ are the square reduced matrix elements whose values are 0.0032, 0.0023, and 0.0002 for λ = 2, 4, and 6. In the case of europium, J = λ^2.

2. Theoretical Radiative Decay Rates

The theoretical radiative decay rate for the forced electric dipole and magnetic dipole governed transitions $A_{rad}^{ed,md}$ is given by $A_{rad}^{ed,md} = A_{rad}^{ed} + A_{rad}^{md}$, where, for europium,

$$A_{rad}^{ed} = \frac{32e^2\pi^3\chi}{3\hbar\times(2J'+1)} \sum_{J=2,4,6} \nu[{}^5D_0 \rightarrow {}^7F_J]^3 \Omega_{\lambda=J}^{calc} \left|\left\langle {}^5D_0 \left\| U^{(\lambda)} \right\| {}^7F_J \right\rangle\right|^2 \quad \text{(S4)}$$

and A_{rad}^{md} is equal to

$$A_{rad}^{md} = \frac{32\pi^3 n^3 \nu[{}^5D_0 \rightarrow {}^7F_1]^3}{3\hbar} S_{md} \quad \text{(S5)}$$

where *J'* is the total angular momentum quantum number of the emitting level, which in the case of europium is ${}^5D_0$ and therefore $J' = 0$. $\nu[{}^5D_0 \rightarrow {}^7F_J]$ in theoretical calculations is usually considered as being the difference between the barycenter of energy of the ${}^5D_0$ and ${}^7F_J$ levels (J = 2, 4, 6), which were determined by Carnall for the trivalent lanthanides in fluoride complexes[2]; and S_{md}, the magnetic dipole strength constant, which, for the trivalent europium ion, is equal to 9.6×10^{-42} $esu^2.cm^2$ [3].

The theoretical intensity parameters Ω_λ^{calc} (λ = 2, 4, 6) emerge from the Judd-Ofelt theory, and are proportional to the intensities of the ${}^5D_0 \rightarrow {}^7F_2$, ${}^5D_0 \rightarrow {}^7F_4$ and ${}^5D_0 \rightarrow {}^7F_6$ transitions, respectively, in the emission spectrum of the complex. These parameters describe the coordination interaction between the lanthanide cation and the ligands, and are given according to the following expression:

$$\Omega_\lambda^{calc} = (2\lambda+1) \sum_{t}^{\lambda-1,\lambda+1(odd)} \sum_{p=-t}^{t(all)} \frac{\left|B_{\lambda tp}\right|^2}{(2t+1)} \quad \text{(S6)}$$

where the $B_{\lambda tp}$ terms are given by the following expression:

$$B_{\lambda tp} = B_{\lambda tp}^{ed} + B_{\lambda tp}^{dc} \quad \text{(S7)}$$

where $B^{ed}_{\lambda tp}$ corresponds to the forced electric dipole contribution and $B^{dc}_{\lambda tp}$ corresponds to the dynamic coupling contribution, given by:

$$B^{ed}_{\lambda tp} = \frac{2}{\Delta E}\left\langle r^{t+1}\right\rangle \theta(t,\lambda)\gamma^t_p \tag{S8}$$

$$B^{dc}_{\lambda tp} = -\left[\frac{(\lambda+1)(2\lambda+3)}{2\lambda+1}\right]^{1/2}\left\langle r^{\lambda}\right\rangle(1-\sigma_{\lambda})\left\langle f\left\|C^{(\lambda)}\right\|f\right\rangle\Gamma^t_p\delta_{t,\lambda+1} \tag{S9}$$

where ΔE is a constant, approximately given by the energy difference between the barycenters of the ground $4f^n$ and first opposite parity excited state of configuration $4f^{(n-1)}5d$ of the europium ion; $\left\langle r^2\right\rangle$ = 2.56754 × 10^{-17}cm^2, $\left\langle r^4\right\rangle$ = 1.58188 × 10^{-33} cm^4, $\left\langle r^6\right\rangle$ = 1.98086 × 10^{-49} cm^6, and $\left\langle r^8\right\rangle$= 6.74786 × 10^{-65} cm^8 are radial integrals pre-defined for the europium ion[4], with $\left\langle r^8\right\rangle$ obtained from extrapolation; θ(t,λ) are numerical factors for a given lanthanide, estimated by Hartree-Fock calculations of the radial integrals[5] as: θ(1,2) =-0.17; θ(3,2) =0.345; θ(3,4) =0.18; θ(5,4) =-0.24; θ(5,6) =-0.24; θ(7,6) =0.24; γ^t_p are the odd-rank ligand field parameters; $(1-\sigma_{\lambda})$ is a shielding factor due to the filled 5s and 5p sub-shells of the lanthanide ion[5], with σ_2 = 0.600, σ_4 = 0.139, and σ_6 = 0.100 for Eu(III); $\left\langle f\left\|C^{(\lambda)}\right\|f\right\rangle$ is a Racah tensor operator of rank λ = 2, 4, and 6 whose value are -1.3660, 1.128, and -1.270, respectively, for any lanthanide; Γ^t_p is also a sum over coordinating atoms which further reflects the chemical environment; finally, $\delta_{t,\lambda+1}$ is a Kronecker delta symbol.

The odd rank ligand field parameters, in turn, are given by:

$$\gamma^t_p = \left(\frac{4\pi}{2t+1}\right)^{\frac{1}{2}} e^2\sum_i \rho_i\left(2\beta_i\right)^{t+1}\frac{g_i}{R_i^{t+1}}Y^{t*}_p(\theta_i,\phi_i) \tag{S10}$$

where i runs over the ligand atoms, ρ_j and β_i are defined according to the Simple Overlap Model (SOM) [6, 7] as

$$\rho_i = \rho_0 \left(\frac{R_0}{R_i} \right)^n \tag{S11}$$

$$\beta_i = \frac{1}{1 \pm \rho_i} \tag{S12}$$

and represent the correction introduced by SOM to the crystal field parameters of PCEM. Indeed, the difference per directly coordinated ligand atom *i*, between the $\gamma_{p,i}^t(SOM)$ of SOM, Eq. (S10) and $\gamma_{p,i}^t(PCEM)$ is

$$\gamma_{p,i}^t(SOM) = \rho_i \left(2\beta_i\right)^{t+1} \gamma_{p,i}^t(PCEM) \tag{S13}$$

In the equations above, ρ_0 is a constant equal to 0.05 for any trivalent lanthanide ion, R_0 is the smallest distance between the lanthanide ion and a directly coordinating atom of the ligand, R_j is the distance between the lanthanide ion and the directly coordinating atom j of the ligand. The plus sign in Eq. (S12) is used when the barycenter of the overlap region is displaced towards the ligand, which happens, usually, in the case of oxygen and fluorine coordinating atoms. The minus sign is used when this barycenter is displaced towards the central ion, as is usually the case of nitrogen and chlorine coordinating atoms. Further, g_i in Eq. (S10) is the charge factor associated to the lanthanide-ligand atom bond; and $Y_p^{t*}(\theta_i, \varphi_i)$ are complex conjugate spherical harmonics, where θ_i and ϕ_i are the angles corresponding to the position of the directly coordinating atom i of the ligands.

The other odd rank parameter Γ_p^t, which further reflects the chemical environment, is given by:

$$\Gamma_p^t = \left(\frac{4\pi}{2t+1} \right)^{\frac{1}{2}} \sum_i \frac{\alpha_i}{R_i^{t+1}} Y_p^{t*}(\theta_i, \phi_i) \tag{S14}$$

where α_i is the polarizability associated to the lanthanide-ligand atom bond.

3. Perturbation theory formalism on the semiempirical wavefunctions

Within the Sparkle Model, the trivalent lanthanide ion is represented by a potential due to the electrostatic point of charge +3e, superimposed to a spherical repulsive potential of the form exp(-αr), in order to prevent the ligands from collapsing into the metal ion. Thus, in this model, the metal ion does not have any orbitals. The ligands, on the other hand, usually constitute a closed shell system described by the wavefunctions of a regular semiempirical model deformed by the potential that represents the metal ion. On the other hand, in the RM1 model for the lanthanides, the metal ion has a semiempirical basis set comprised of 5d, 6s, and 6p atomic orbitals. In both cases, the 4f electrons are considered part of the core, and are therefore taken care of implicitly. Accordingly, within these models, the restricted Hartree-Fock ground state wavefunction for the closed shell complex of 2n electrons is represented by a single Slater determinant:

$$\Psi(1,2,...,2n) = \frac{1}{\left|(2n!)^{1/2}\right|}\left|\psi_1(1)\overline{\psi}_1(2)...\psi_n(2n-1)\overline{\psi}_n(2n)\right| \tag{S15}$$

where the bars on top indicate a beta spin and ψ_i are normalized molecular orbitals, which can be expressed in terms of a linear combination of semiempirical atomic orbitals χ_p^μ as:

$$\psi_i = \sum_\mu^{N_A}\sum_p^{N_\mu} c_{pi}^\mu \chi_p^\mu \tag{S16}$$

where μ runs over the N_A atoms, p runs over the N_μ atomic orbitals of atom μ and c_{pi}^μ is the linear coefficient of the semiempirical atomic orbital χ_p^μ in the molecular orbital of order i.

The corresponding Hartree-Fock ground state energy is:

$$E_{ele} = 2\sum_i^n H_{ii} + \sum_i^n\sum_j^n (2J_{ij} - K_{ij}) \tag{S17}$$

where H_{ii} is the core energy of the ith molecular orbital:

$$H_{ii} = \langle \psi_i(1) \left| H^{core} \right| \psi_i(1) \rangle \tag{S18}$$

with

$$H^{core} = -\frac{1}{2}\nabla_1^2 - \sum_{\mu}^{N_A} \frac{Z_\mu}{r_{\mu 1}} \tag{S19}$$

where Z_μ is the atomic number of atom μ, and $r_{\mu 1}$ is the distance of electron 1 to the center of atom μ.

Applying Eq. (S16) in Eq. (S18), we obtain

$$H_{ii} = \sum_{\mu}^{N_A} \sum_{p}^{N_\mu} \sum_{\nu}^{N_A} \sum_{q}^{N_\nu} c_{pi}^{\mu} c_{qi}^{\nu} \left\langle \chi_p^\mu \left| H^{core} \right| \chi_q^\nu \right\rangle \tag{S20}$$

which can be partitioned as:

$$H_{ii} = \sum_{\mu}^{N_A} \alpha'_\mu + \sum_{\mu}^{N_A} \sum_{\nu}^{N_A} \beta_{\mu\nu} \tag{S21}$$

where α'_μ and β_ν can be defined as:

$$\alpha'_\mu = \sum_{p}^{N_\mu} \sum_{q}^{N_\mu} c_{pi}^{\mu} c_{qi}^{\mu} \left\langle \chi_p^\mu \left| H^{core} \right| \chi_q^\mu \right\rangle \tag{S22}$$

$$\beta_{\substack{\mu\nu \\ \mu \neq \nu}} = \sum_{p}^{N_\mu} \sum_{q}^{N_\nu} c_{pi}^{\mu} c_{qi}^{\nu} \left\langle \chi_p^\mu \left| H^{core} \right| \chi_q^\nu \right\rangle \tag{S23}$$

α'_μ can be interpreted as the energy of an electron in the atomic orbitals of atom μ, being attracted by the nuclei of all atoms of the molecule. Likewise, $\beta_{\mu\nu}$ can be seen as the binding ability between atoms μ and ν.

When either the Sparkle Model or the RM1 model for lanthanides calculations are usually carried out, we obtain the geometry of the complex in its minimum and in its electronic singlet ground state. The luminescence involves absorption of UV photons by their ligands, which go to an excited singlet state, and then, subsequently, go to a lower

energy triplet state. The absorbed energy is then transferred to the metal ion which goes to an excited state - in the case of the europium ion, to the $^5D_0$ state, while the ligands are back to their singlet ground state and the geometry of the complex is, for all practical purposes, the same as the initial one calculated for the complex. Now, what must occur is the decay of the metal ion from its excited state ($^5D_0$ for europium(III)) to lower energy level ones. This decay may take two forms: radiatively (from $^5D_0$ to ^7F_J, J = 0 to 6 for europium) and nonradiatively. Each form is described by its corresponding decay rates, either A_{rad} and A_{nrad}. It is the radiative decay process, which is kinetically governed by $A_{rad} = A_{rad}^{ed} + A_{rad}^{md}$ which is the one that we are concerned with, via Eqs. (S4) and (S5). The almost essentially electrostatic coordinated bond between the ligands and the metal ion, can be viewed both as a perturbation on the ligands by the metal ion, and as a perturbation on the metal ion by the ligands, making luminescence possible. Likewise, the ligands perturb the metal ion, which makes the radiative process a phenomenon which occurs with the concurrence of the ligands, and more intensely so, with the partnership of their directly coordinated atoms. This cooperation is therefore important in the luminescence process as it is included into $A_{rad} = A_{rad}^{ed} + A_{rad}^{md}$ in the form of parameters g_i and α_i, which reflect this partnership, as they affect the value of A_{rad}. In this work, as stated in the main article, we model g_i and α_i as resulting from a perturbation by the metal ion on the Sparkle Model or RM1 model for the lanthanides wavefunctions of the ligands.

We will now proceed with deriving the perturbation theory formulas in a way previously introduced by one of us [8], which will describe the effect of the lanthanide trivalent ion on the coordinating atoms of the ligands as a perturbation.

Accordingly, assuming that Ψ(1,2,…,2n) is the zeroth order wavefunctions, from now on represented as $\Psi_k^{(0)}(1,2,...,2n)$, obtained from the solution of the Hartree-Fock

equations as $H^{(0)}\Psi_k^{(0)} = E_k^{(0)}\Psi_k^{(0)}$, where the ground state function, the one that is usually computed by the semiempirical methods, is represented by k = 1.

As excited state wavefunctions to be considered in the perturbation formulae, we will only take into account those singlet states obtained from single excitations of the ground state determinant[8], represented by

$$\Psi_i^a = \frac{1}{|2(2n!)|^{1/2}} \left|\psi_1\overline{\psi}_1 \ldots \overline{\psi}_{i-1}\right|\psi_i\overline{\psi}_a - \overline{\psi}_i\psi_a\left|\psi_{i+1}\ldots\overline{\psi}_n\right| \qquad \text{(S24)}$$

The electronic energies associated with these functions can be obtained from:

$$E_g^{(0)} = \left\langle \Psi_g^{(0)} \middle| H^{(0)} \middle| \Psi_g^{(0)} \right\rangle \qquad \text{(S25)}$$

$$E_i^a = \left\langle \Psi_i^a \middle| H^{(0)} \middle| \Psi_i^a \right\rangle \qquad \text{(S26)}$$

We will also assume that Koopmans theorem is strictly valid and we will therefore take as the energy difference between the ground state $\Psi_g^{(0)}$ and the excited state Ψ_i^a, the difference in the energies of the molecular orbitals ψ_i and ψ_a as $E_g^{(0)} - E_i^a = \varepsilon_i - \varepsilon_a$, an approximation that is accurate enough for our purposes.

Now, assume that a perturbation $H^{(1)}$, which can be described as a sum of one-electron operators, Θ_l, affects the system:

$$H = H^{(0)} + H^{(1)} \qquad \text{(S27)}$$

$$H^{(1)} = \sum_{l=1}^{2n} \Theta_l \qquad \text{(S28)}$$

The first order energy becomes

$$E_g^{(1)} = \left\langle \Psi_g^{(0)} \middle| H^{(1)} \middle| \Psi_g^{(0)} \right\rangle = 2\sum_{i=1}^{n} \left\langle \psi_i \middle| \Theta_1 \middle| \psi_i \right\rangle \qquad \text{(S29)}$$

In order to obtain the second order correction to the energy, it is necessary to obtain the first order correction to the wave function. From perturbation theory,

$$\Psi_g^{(1)} = \Psi_g^{(0)} + \sum_{i}^{occ} \sum_{a}^{unocc} \frac{\left\langle \Psi_i^a \middle| H^{(1)} \middle| \Psi_g^{(0)} \right\rangle}{\varepsilon_i - \varepsilon_a} \Psi_i^a \tag{S30}$$

where the sum in i runs over all occupied orbitals and the sum in a runs over all unoccupied ones.

It is easy to show that

$$\left\langle \Psi_i^a \middle| H^{(1)} \middle| \Psi_g^{(0)} \right\rangle = \sqrt{2} \left\langle \psi_a \middle| \Theta_1 \middle| \psi_i \right\rangle \tag{S31}$$

The first-order wavefunction then becomes:

$$\Psi_g^{(1)} = \Psi_g^{(0)} + \sqrt{2} \sum_{i}^{occ} \sum_{a}^{unocc} \frac{\left\langle \psi_a \middle| \Theta_1 \middle| \psi_i \right\rangle}{\varepsilon_i - \varepsilon_a} \Psi_i^a \tag{S32}$$

The second order energy is defined as

$$E_g^{(2)} = \left\langle \Psi_g^{(0)} \middle| H^{(1)} \middle| \Psi_g^{(1)} \right\rangle \tag{S33}$$

Applying Eq. (S32) into Eq. (S33),

$$E_g^{(2)} = \sqrt{2} \sum_{i}^{occ} \sum_{a}^{unocc} \frac{\left\langle \psi_a \middle| \Theta_1 \middle| \psi_i \right\rangle \left\langle \Psi_g^{(0)} \middle| H^{(1)} \middle| \Psi_i^a \right\rangle}{\varepsilon_i - \varepsilon_j} \tag{S34}$$

and applying Eq. (S31) into Eq. (S34),

$$E_g^{(2)} = 2 \sum_{i}^{occ} \sum_{a}^{unocc} \frac{\left\langle \psi_a \middle| \Theta_1 \middle| \psi_i \right\rangle \left\langle \psi_i \middle| \Theta_1 \middle| \psi_a \right\rangle}{\varepsilon_i - \varepsilon_a} \tag{S35}$$

Now, we can express the formulas for the first and second order energies in terms of the atomic orbitals by applying Eq. (S16) into Eq. (S29) and Eq. (S35).

$$E_g^{(1)} = 2 \sum_{i} \sum_{\mu} \sum_{p} \sum_{\nu} \sum_{q} c_{pi}^{\mu} c_{qi}^{\nu} \left\langle \chi_p^{\mu} \middle| \Theta_1 \middle| \chi_q^{\nu} \right\rangle \tag{S36}$$

$$E_g^{(2)} = 2 \sum_{i} \sum_{a} \sum_{\lambda} \sum_{r} \sum_{\mu} \sum_{p} \sum_{\nu} \sum_{q} \sum_{\sigma} \sum_{s} c_{ra}^{\lambda} c_{pi}^{\mu} c_{qi}^{\nu} c_{sa}^{\sigma} \frac{\left\langle \chi_r^{\lambda} \middle| \Theta_1 \middle| \chi_p^{\mu} \right\rangle \left\langle \chi_q^{\nu} \middle| \Theta_1 \middle| \chi_s^{\sigma} \right\rangle}{\varepsilon_i - \varepsilon_a} \tag{S37}$$

where i runs over the occupied orbitals, and a runs over the unoccupied ones; and r, p, q, and s run over the atomic orbitals of all atoms λ, μ, ν, and σ of the ligands.

4. g_i charges from the semiempirical models

In order to model the effect by the metal ion on the directly coordinated atoms of the ligands, as before[8], we will use an empirical perturbation. Since the metal ion is charged, the modification of the electron density of the metal should most affect the α'_μ as defined by Eq. (S22). Further, assuming that the perturbation is only affecting atom μ, we will assume that there will be only variations in α'_μ. That is, if we do not consider the perturbation as a vector quantity, we can represent it as:

$$\left\langle \chi_r^\lambda \middle| \Theta_1 \middle| \chi_s^\sigma \right\rangle = \delta\alpha'_\mu . \delta_{\lambda\sigma} . \delta_{rs} \qquad \text{(S38)}$$

Applying this Eq. (S38) into Eq. (S36), we can calculate the first order correction of the electronic energy as:

$$E_g^{(1)} = 2\sum_i^n \sum_p^{N_\mu} \left| c_{pi}^\mu \right|^2 \delta\alpha'_\mu \qquad \text{(S39)}$$

And therefore,

$$E_g^{(1)} = q_\mu \delta\alpha'_\mu \qquad \text{(S40)}$$

where q_μ is the ZDO electronic density of atom μ, and $\delta\alpha'_\mu$ is a measure of the extent of the perturbation.

So, in this work, we postulate that the charge factors g_i in Eq. (S10) is homomorphic with Eq. (S40) and equal to

$$g_i = Q . q_i \qquad \text{(S41)}$$

where Q is a single parameter to be applied to all ZDO electronic densities of all directly coordinated atoms, adjusted in order to reproduce the experimentally obtained $\Omega_2^{\exp}$ and

$\Omega_4^{\exp}$. Please, note that the index i used in g_i and Eq. (S41) is consistent with the notation traditionally used in lanthanide luminescence theory and should not be confused with index i of the perturbation theory formalism on the semiempirical wavefunction, where it denotes an occupied molecular orbital, as in Eq. (S39).

5. α_i polarizabilities from the semiempirical models

We now proceed to further model the effect by the metal ion on the directly coordinated atoms of the ligands, as a second order effect. So, we start by assuming that the complex is composed of two systems: the ligands with their atoms in their positions, represented by L, and the metal ion represented by M, and that each of these two systems obeys their corresponding eigenvalue equations as:

$$H^{(0)L}\Psi_l^{(0)L} = E_l^{(0)L}\Psi_l^{(0)L} \qquad \text{(S42)}$$

$$H^{(0)M}\Psi_m^{(0)M} = E_m^{(0)L}\Psi_m^{(0)M} \qquad \text{(S43)}$$

We now assume that no energy level of the ligands, $E_l^{(0)L}$, is identical to any of the energy levels of the metal, $E_m^{(0)M}$. We now further assume that the metal ion will interact independently with each of the directly coordinating atoms of the ligands. Thus, assuming that any atomic orbital of the ligands can interact with any atomic orbital of the metal, this empirical perturbation can be represented[8] by:

$$\left\langle \chi_r^{\lambda} \middle| \Theta_1 \middle| \chi_s^{\varphi} \right\rangle = \delta\beta_{\sigma\tau}(\delta_{\sigma\lambda}.\delta_{\tau\varphi} + \delta_{\sigma\varphi}.\delta_{\tau\lambda}) \qquad \text{(S44)}$$

where λ represents any atom belonging to the ligands; τ represents the metal ion, and ϕ represents any atoms which may belong to any of the two systems: ligands and metal.

As the perturbation defined above (Eq. (S44)) does not affect each of the two systems, L and M, taken independently, the first order correction to the energies, obtained from Eq. (S29), can be easily proven to be zero:

$$E_g^{(1)L} = 2\sum_i^{occ.} \left\langle \psi_i^L \left| \Theta_1 \right| \psi_i^L \right\rangle = 0 \tag{S45}$$

$$E_g^{(1)M} = 2\sum_i^{occ.} \left\langle \psi_j^M \left| \Theta_1 \right| \psi_j^M \right\rangle = 0 \tag{S46}$$

In order to obtain the second order correction to the energies, we need to obtain the first order correction to the each of the wavefunctions: the one of the ligands and the other of the metal ion. We will now use *i* as the index of the occupied orbitals of the ligands and *j* as the index of the occupied orbitals of the metal ion. The summation over a is over the unoccupied orbitals of the ligands and the summation over b is over the unoccupied orbitals of the metal ion. For the purpose of this work, we will consider as excited states of the ligands, not only Ψ_i^a, but also Ψ_i^b, when an electron goes from the occupied orbital of the ligands, ψ_i^L, to an unoccupied orbital of the metal ion, ψ_b^M, with these two levels in singlet coupling. For the metal ion, M, we will also take into account, besides Ψ_j^b, also Ψ_j^a. These states Ψ_i^b and Ψ_j^a are charge density transfer states.

Accordingly, the first order correction to the wavefunctions of the ligands and of the metal are:

$$\Psi_g^{(1)L} = \sqrt{2}\sum_i \sum_a \frac{\left\langle \psi_a^L \left| \Theta_1 \right| \psi_i^L \right\rangle}{\varepsilon_i - \varepsilon_a} \Psi_i^a + \sqrt{2}\sum_i \sum_b \frac{\left\langle \psi_b^M \left| \Theta_1 \right| \psi_i^L \right\rangle}{\varepsilon_i - \varepsilon_b} \Psi_i^b \tag{S47}$$

$$\Psi_g^{(1)M} = \sqrt{2}\sum_j \sum_b \frac{\left\langle \psi_j^M \left| \Theta_1 \right| \psi_b^M \right\rangle}{\varepsilon_j - \varepsilon_b} \Psi_j^b + \sqrt{2}\sum_j \sum_a \frac{\left\langle \psi_a^L \left| \Theta_1 \right| \psi_j^M \right\rangle}{\varepsilon_j - \varepsilon_a} \Psi_j^a \tag{S48}$$

The terms that possess integrals over levels of the same molecule are zero for the same reason that the first order correction to the energies are zero. As such, applying Eqs. (S47) and (S48) into Eq.(S33), we obtain

$$E_g^{(2)L} = 2\sum_i \sum_b \frac{\left\langle \psi_b^M \left| \Theta_1 \right| \psi_i^L \right\rangle \left\langle \psi_i^L \left| \Theta_1 \right| \psi_b^M \right\rangle}{\varepsilon_i - \varepsilon_b} \tag{S49}$$

$$E_g^{(2)M} = 2\sum_j \sum_a \frac{\left\langle \psi_a^L \left| \Theta_1 \right| \psi_j^T \right\rangle \left\langle \psi_j^M \left| \Theta_1 \right| \psi_a^L \right\rangle}{\varepsilon_j - \varepsilon_a} \tag{S50}$$

By replacing the molecular orbitals by their respective expansion in terms of atomic orbitals, we now have

$$E_g^{(2)L} = 2\sum_i \sum_b \sum_r \sum_p \sum_q \sum_m \frac{c_{rb}^{\tau} c_{pi}^{\sigma} c_{qi}^{\sigma} c_{mb}^{\tau}}{\varepsilon_i - \varepsilon_b} (\delta\beta_{\sigma\tau})^2 \tag{S51}$$

$$E_g^{(2)M} = 2\sum_j \sum_a \sum_p \sum_r \sum_m \sum_q \frac{c_{rb}^{\sigma} c_{pi}^{\tau} c_{qi}^{\tau} c_{mb}^{\sigma}}{\varepsilon_j - \varepsilon_a} (\delta\beta_{\sigma\tau})^2 \tag{S52}$$

The total interaction energy is the sum of both corrections

$$E_g^{(2)L+M} = E_g^{(2)L} + E_g^{(2)M} \tag{S53}$$

At this point, we must introduce into the model a postulate that the metal ion is well defined and does not modify itself to adapt to the chemical environment – reflecting the experimental fact that it is relatively insensitive to the environment. So, we will first assume that the second order correction to the metal ion will be a constant, albeit different for each different complex, $E_g^{(2)M}$. Then, we will further assume that since the metal ion is a trivalent species, its dominant interaction with the ligands will be electrophilic, that is, with the occupied orbitals of the ligands. Therefore, we will restrict our sum over index i to the occupied orbitals of the ligands. Moreover, in the case of the Sparkle Model, since orbitals are non-existent, there will be no explicit orbital energy and no linear coefficients. We will thus postulate, as in the previous work by Simas[8], for the purpose of using Eq. (S51) within both the Sparkle Model and the RM1 model for the lanthanides, that ε_b is non-existent, and zeroed, and the product of the coefficients of the orbitals of the metal, $c_{rb}^{\tau} c_{mb}^{\tau}$ is equal to 1.

The total interaction energy will now be:

$$E_g^{(2)L+M} = E_g^{(2)M} + 2\sum_i^{occ.} \sum_p \sum_q \frac{c_{pi}^{\sigma} c_{qi}^{\sigma}}{\varepsilon_i} (\delta\beta_{\sigma\tau})^2 \tag{S54}$$

As a generalization to an all valence electron method of the corresponding superdelocalizability of Fukui[9], and as originally introduced by Simas[8], we now call the multiplying factor to $(\delta\beta_{\sigma\tau})^2$ the electrophilic superdelocalizability of atom σ, defined as

$$SE_{\sigma} = 2\sum_{i}^{occ.}\sum_{p}\sum_{q}\frac{c_{pi}^{\sigma}c_{qi}^{\sigma}}{\varepsilon_i} \tag{S55}$$

In this sense, our electrophilic superdelocalizability[8] is unique and differs from the one in the article by Lewis[10] and also in the article by Brown and Simas[11] because in these articles the authors do not take into account the cross-products of the atomic orbitals for each molecular orbital. And it also differs even more from the delocalizability of Schüürmann[12, 13], $D^E(i)$, because not only, as Lewis[10] and as Simas an Brown[11], he does not take into account the cross-products of the atomic orbitals for each molecular orbital, but also because, instead, he uses a different denominator:

$$D_{\sigma}^{E} = 2\sum_{i}^{occ}\sum_{p}\frac{\left(c_{pi}^{\sigma}\right)^2}{\varepsilon_i - \frac{\left(\varepsilon_{HOMO}+\varepsilon_{LUMO}\right)}{2}} \tag{S56}$$

As we carried out research for this article, we also tried to use the superdelocalizability of Lewis[10] and of Simas and Brown[11], and also the delocalizability of Schüürmann, but they all did not produce good fittings. So, we stayed with the superdelocalizability as defined by Eq. (S55) above.

Hence, we will use as the polarizability α_i in Eq. (S54), an expression homomorphic with Eq. (S54), a first degree polynomial:

$$\alpha_i = SE_i \cdot D + C \tag{S57}$$

with the constants *D* and *C* being the same for all directly coordinated atoms *i* and adjusted in order to reproduce the various experimentally obtained $\Omega_{\lambda}^{\exp}$ with λ = 2,4. Once again, please note that index i of Eq. (S57) above refers to a directly coordinated atom in agreement with the usual notation employed by lanthanide luminescence theory, and should not be confused with index i of the semiempirical perturbation theory, as in Eqs. (S54) to (S56).

References

1. Jørgensen, C.K. & Judd, B.R. Hypersensitive pseudoquadrupole transitions in lanthanides. *Mol. Phys.* **8**, 281-290 (1964).
2. Carnall, W.T., Crosswhite, H. & Crosswhite, H.M. in Argonne National Laboratory Report (1977).
3. Weber, M.J., Varitimo.Te & Matsinge.Bh Optical Intensities of Rare-Earth Ions in Yttrium Orthoaluminate. *Phys. Rev. B* **8**, 47-53 (1973).
4. Freeman, A.J. & Desclaux, J.P. Dirac-Fock Studies of Some Electronic Properties of Rare-Earth Ions. *J. Magn. Magn. Mater.* **12**, 11-21 (1979).
5. Malta, O.L., Ribeiro, S.J.L., Faucher, M. & Porcher, P. Theoretical Intensities of 4f-4f Transitions between Stark Levels of the Eu3+ Ion in Crystals. *J. Phys. Chem. Solids* **52**, 587-593 (1991).
6. Malta, O.L. A Simple Overlap Model in Lanthanide Crystal-Field Theory. *Chem. Phys. Lett.* **87**, 27-29 (1982).
7. Malta, O.L. Theoretical Crystal-Field Parameters for the Yoc1 - Eu-3+ System - A Simple Overlap Model. *Chem. Phys. Lett.* **88**, 353-356 (1982).
8. Simas, A.M. in Instituto de Química, Vol. Master of Sciences Dissertation (Universidade Estadual de Campinas, Campinas, Brazil; 1977).
9. Fukui, K., Yonezawa, T., Nagata, C. & Shingu, H. Molecular Orbital Theory of Orientation in Aromatic, Heteroaromatic, and Other Conjugated Molecules. *J. Chem. Phys.* **22**, 1433-1442 (1954).
10. Lewis, D.F.V. Molecular orbital calculations on solvents and other small molecules: Correlation between electronic and molecular properties ν, αMOL, π*, and β. *J. Comput. Chem.* **8**, 1084-1089 (1987).
11. Brown, R.E. & Simas, A.M. On the Applicability of Cndo Indexes for the Prediction of Chemical-Reactivity. *Theor. Chim. Acta* **62**, 1-16 (1982).
12. Schüürmann, G. QSAR analysis of the acute fish toxicity of organic phosphorothionates using theoretically derived molecular descriptors. *Environ. Toxicol. Chem.* **9**, 417-428 (1990).
13. Schüürmann, G. in Rational Approaches to Structure, Activity, and Ecotoxicology of Agrochemicals, Edn. 1 edition. (eds. T. Fujita & W. Draber) 485-541 (CRC Press, Florida, USA; 1992).

Additional Figures and Tables

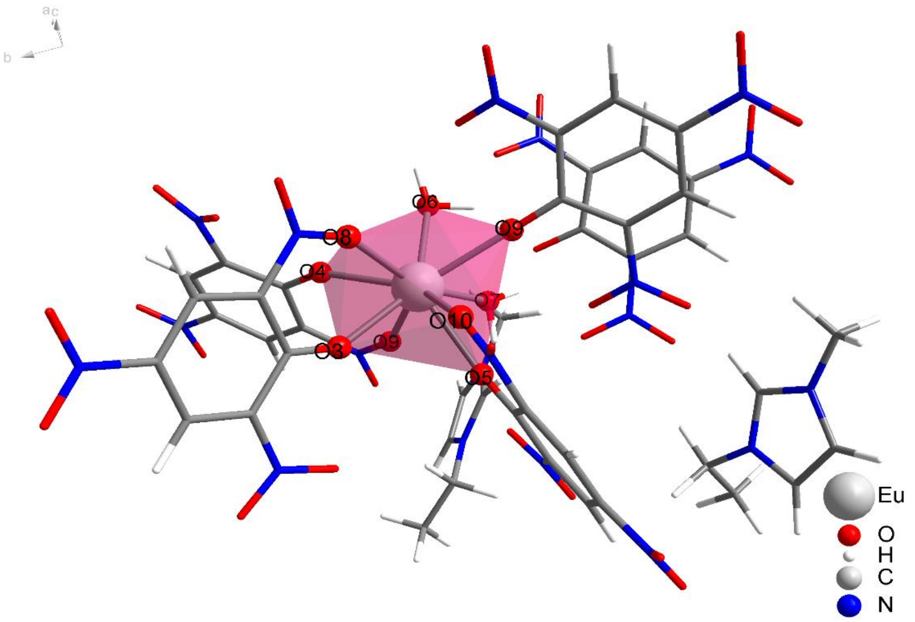

Figure S1. Perspective view of the crystallographic geometry of complex **(EMIm)$_2$[Eu(PIC)$_4$(H$_2$O)$_2$]PIC**, CSD deposited entry 854429.

Table S1. Sparkle/RM1 and RM1 model for Eu(III) ZDO electronic densities *q* and electrophilic superdelocalizabilities *SE* for each atom directly coordinated to europium(III), in complex **(EMIm)$_2$[Eu(PIC)$_4$(H$_2$O)$_2$]PIC**, CSD deposited entry 854429, at the crystallographic geometry, together with corresponding charge factors *g* and polarizabilities α from the fitting.

Ligand Atom	**Sparkle/RM1**				**RM1 model for Eu(III)**			
	Q = 0.0454 au^{-1}<br>D = 19.9 au^{-1}·Å^3<br>C = 11.1 Å^3<br>D/C = 1.79 au^{-1}				Q = 0.0442 au^{-1}<br>D = 23.3 au^{-1}·Å^3<br>C = 11.6 Å^3<br>D/C = 2.00 au^{-1}			
	q (au)	*SE* (au)	*g*	α (Å^3)	*q* (au)	*SE* (au)	*g*	α (Å^3)
O2 (PIC1)	6.71	-0.491	0.305	1.35	6.39	-0.440	0.283	1.39
O3 (PIC2)	6.69	-0.368	0.304	3.78	6.36	-0.333	0.282	3.87
O4 (PIC3)	6.66	-0.439	0.302	2.39	6.34	-0.409	0.281	2.10
O5 (PIC4)	6.66	-0.382	0.302	3.51	6.36	-0.333	0.282	3.88
O6 (H_2O)	6.58	-0.282	0.299	5.50	6.28	-0.266	0.278	5.42
O7 (H_2O)	6.60	-0.425	0.300	2.66	6.32	-0.378	0.280	2.82
O8 (PIC2)	6.60	-0.331	0.300	4.54	6.30	-0.309	0.279	4.44
O9 (PIC3)	6.56	-0.419	0.298	2.77	6.30	-0.385	0.279	2.67
O10 (PIC4)	6.55	-0.384	0.297	3.47	6.29	-0.359	0.278	3.27

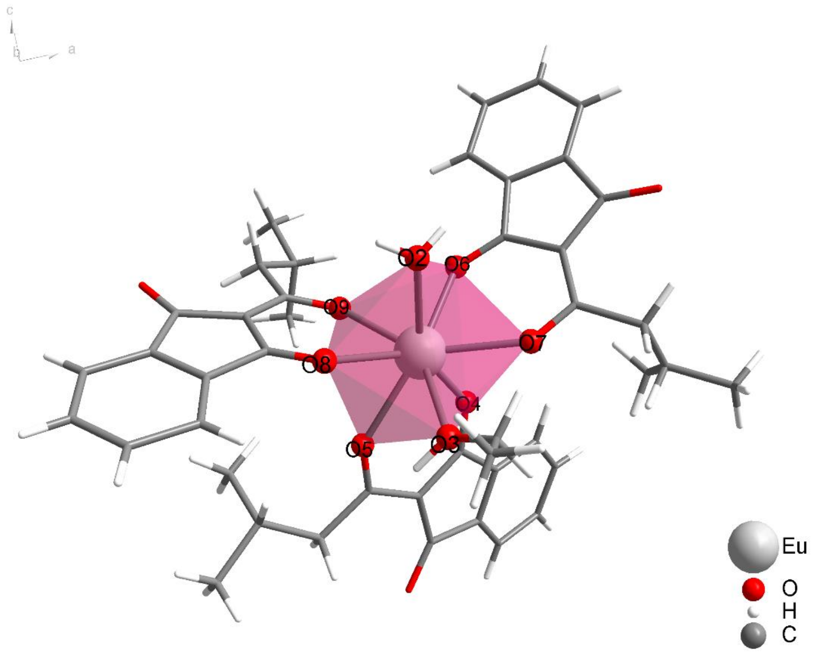

Figure S2. Perspective view of the crystallographic geometry of complex **Eu(ISOVIND)$_3$(H$_2$O)(EtOH)**, CSD code **DEVHOC**.

Table S2. Sparkle/RM1 and RM1 model for Eu(III) ZDO electronic densities *q* and electrophilic superdelocalizabilities *SE* for each atom directly coordinated to europium(III), in complex **Eu(ISOVIND)$_3$(H$_2$O)(EtOH)**, CSD code **DEVHOC**, at the crystallographic geometry, together with corresponding charge factors *g* and polarizabilities α from the fitting.

Ligand Atom	**Sparkle/RM1** Q = 0.0338 au^{-1} D = 38.6 au^{-1}·Å^3 C = 19.7 Å^3 D/C = 1.96 au^{-1}				**RM1 model for Eu(III)** Q = 0.0292 au^{-1} D = 39.8 au^{-1}·Å^3 C = 18.5 Å^3 D/C = 2.15 au^{-1}			
	q (au)	*SE* (au)	*g*	α (Å^3)	*q* (au)	*SE* (au)	*g*	α (Å^3)
O2 (H$_2$O)	6.53	-0.246	0.221	10.2	6.25	-0.237	0.182	9.08
O3 (EtOH)	6.58	-0.412	0.222	3.78	6.31	-0.368	0.184	3.85
O4 (ISOVIND1)	6.67	-0.475	0.225	1.35	6.31	-0.433	0.184	1.28
O5 (ISOVIND1)	6.74	-0.474	0.228	1.39	6.38	-0.389	0.186	3.04
O6 (ISOVIND2)	6.67	-0.316	0.225	7.47	6.33	-0.299	0.185	6.63
O7 (ISOVIND2)	6.77	-0.243	0.229	10.3	6.40	-0.222	0.187	9.68
O8 (ISOVIND3)	6.71	-0.474	0.227	1.36	6.37	-0.398	0.186	2.66
O9 (ISOVIND3)	6.73	-0.500	0.228	0.384	6.35	-0.449	0.185	0.629

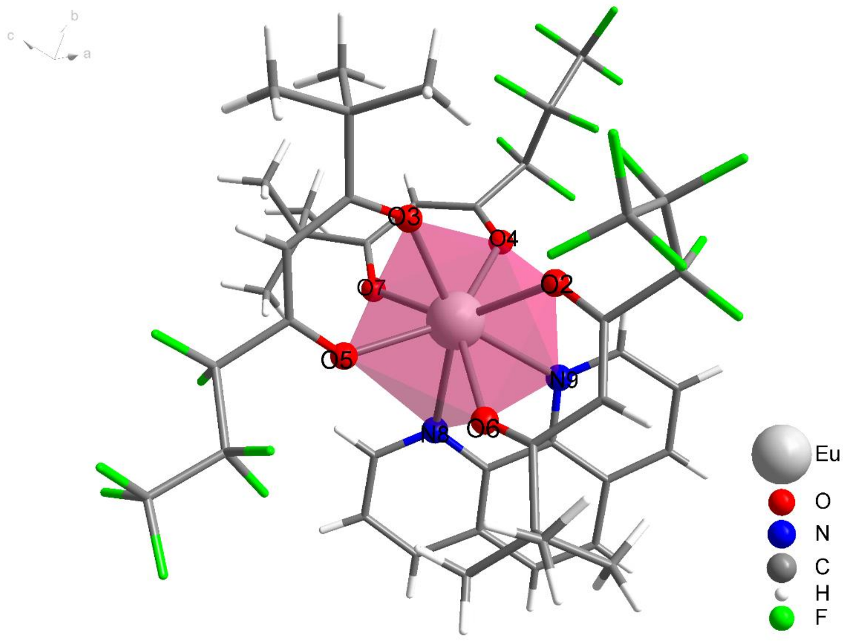

Figure S3. Perspective view of the crystallographic geometry of complex **Eu(FOD)$_3$(PHEN)**, CSD code **EWOCOJ**.

Table S3. Sparkle/RM1 and RM1 model for Eu(III) ZDO electronic densities *q* and electrophilic superdelocalizabilities *SE* for each atom directly coordinated to europium(III), in complex **Eu(FOD)$_3$(PHEN)**, CSD code **EWOCOJ**, at the crystallographic geometry, together with corresponding charge factors *g* and polarizabilities α from the fitting.

Ligand Atom	**Sparkle/RM1**<br><br>Q = 0.260 au^{-1}<br>D = 33.3 au^{-1}·Å^3<br>C = 16.7 Å^3<br>D/C = 1.99 au^{-1}				**RM1 model for Eu(III)**<br><br>Q = 0.314 au^{-1}<br>D = 36.6 au^{-1}·Å^3<br>C = 16.7 Å^3<br>D/C = 2.19 au^{-1}			
	q (au)	*SE* (au)	*g*	α (Å^3)	*q* (au)	*SE* (au)	*g*	α (Å^3)
O2 (FOD1)	6.72	-0.502	1.74	0.0052	6.34	-0.456	2.00	0.0051
O3 (FOD2)	6.72	-0.439	1.74	2.10	6.33	-0.399	1.99	2.11
O4 (FOD3)	6.71	-0.437	1.74	2.15	6.32	-0.382	1.99	2.75
O5 (FOD2)	6.73	-0.348	1.75	5.11	6.35	-0.297	2.00	5.84
O6 (FOD1)	6.60	-0.385	1.71	3.90	6.27	-0.336	1.97	4.42
O7 (FOD3)	6.71	-0.384	1.74	3.94	6.35	-0.327	2.00	4.73
O8 (PHEN)	5.49	-0.466	1.42	1.18	5.21	-0.380	1.64	2.80
O9 (PHEN)	5.48	-0.314	1.42	6.26	5.20	-0.270	1.64	6.83

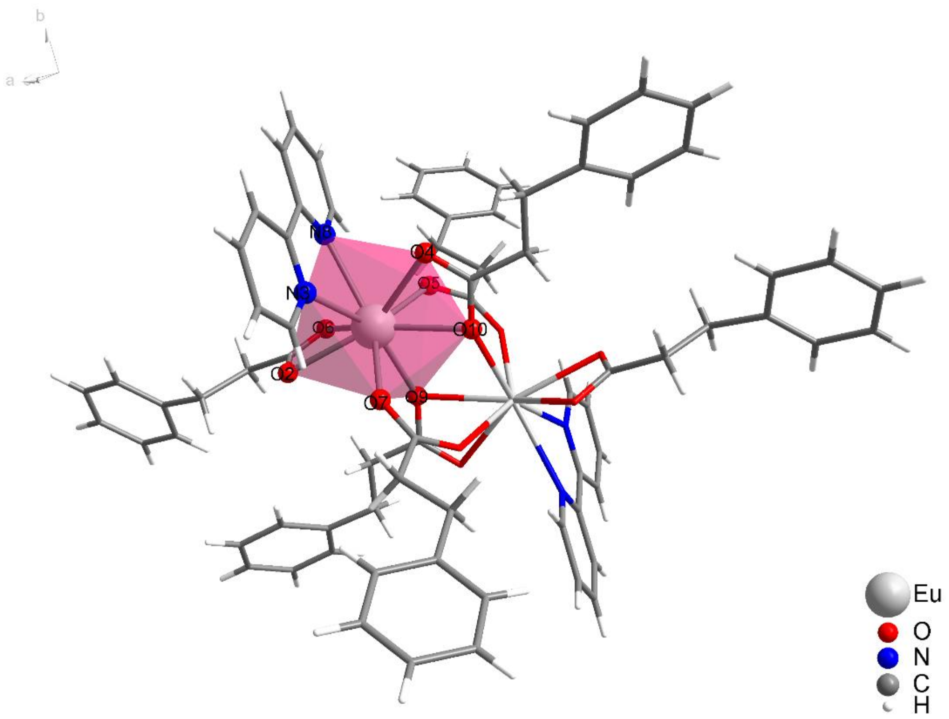

Figure S4. Perspective view of the crystallographic geometry of complex **$Eu_2(CYN)_6(BPY)_2$**, CSD code **LOLXAN**.

Table S4. Sparkle/RM1 and RM1 model for Eu(III) ZDO electronic densities *q* and electrophilic superdelocalizabilities *SE* for each atom directly coordinated to europium(III), in complex **$Eu_2(CYN)_6(BPY)_2$**, CSD code **LOLXAN**, at the crystallographic geometry, together with corresponding charge factors *g* and polarizabilities α from the fitting.

Ligand Atom	**Sparkle/RM1**<br>Q = 0.00416 au^{-1}<br>D = 16.4 au^{-1}·Å^3<br>C = 10.9 Å^3<br>D/C = 1.50 au^{-1}				**RM1 model for Eu(III)**<br>Q = 0.0435 au^{-1}<br>D = 25.2 au^{-1}·Å^3<br>C = 13.0 Å^3<br>D/C = 1.93 au^{-1}			
	q (au)	*SE* (au)	*g*	α (Å^3)	*q* (au)	*SE* (au)	*g*	α (Å^3)
O2 (CIN1)	6.70	-0.466	0.279	3.26	6.39	-0.410	0.278	2.70
N3 (BPY)	5.46	-0.269	0.227	6.51	5.20	-0.255	0.226	6.60
O4 (CIN)	6.62	-0.363	0.275	4.96	6.32	-0.311	0.275	5.19
O5 **(CIN)**	6.76	-0.444	0.281	3.64	6.40	-0.379	0.279	3.48
O6 (CIN1)	6.70	-0.436	0.279	3.76	6.39	-0.386	0.278	3.32
O7 **(CIN)**	6.79	-0.400	0.283	4.35	6.41	-0.340	0.279	4.46
N8 (BPY)	5.48	-0.269	0.228	6.50	5.20	-0.256	0.226	6.59
O9 (CIN)	6.93	-0.488	0.288	2.90	6.39	-0.394	0.278	3.12
O10 (CIN)	6.93	-0.443	0.288	3.64	6.39	-0.350	0.278	4.22

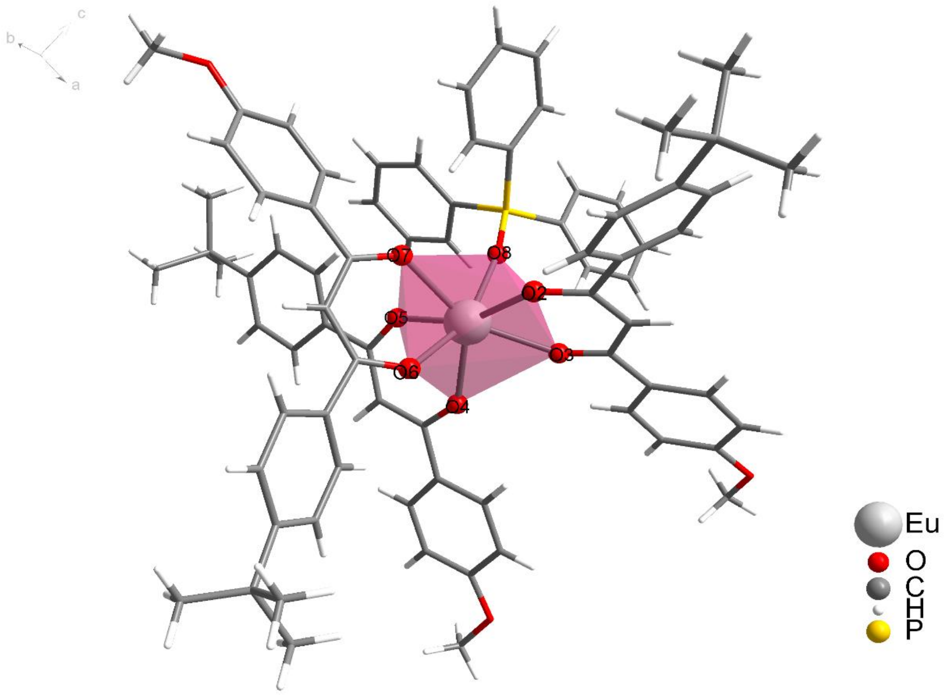

Figure S5. Perspective view of the crystallographic geometry of complex **Eu(BMDM)₃(TPPO)**, CSD code **OTOYEC**.

Table S5. Sparkle/RM1 and RM1 model for Eu(III) ZDO electronic densities *q* and electrophilic superdelocalizabilities *SE* for each atom directly coordinated to europium(III), in complex **$Eu(BMDM)_3(TPPO)$**, CSD code **OTOYEC**, at the crystallographic geometry, together with corresponding charge factors *g* and polarizabilities α from the fitting.

Ligand Atom	**Sparkle/RM1**<br>Q = 0.271 au^{-1}<br>D = 58.0 au^{-1}·Å^3<br>C = 29.2 Å^3<br>D/C = 1.99 au^{-1}				**RM1 model for Eu(III)**<br>Q = 0.292 au^{-1}<br>D = 68.4 au^{-1}·Å^3<br>C = 30.8 Å^3<br>D/C = 2.22 au^{-1}			
	q (au)	*SE* (au)	*g*	α (Å^3)	*q* (au)	*SE* (au)	*g*	α (Å^3)
O2 (BMDM1)	6.73	-0.461	1.82	2.43	6.34	-0.387	1.85	4.31
O3 (BMDM1)	6.75	-0.503	1.83	0.0056	6.34	-0.450	1.85	0.0058
O4 (BMDM2)	6.75	-0.457	1.83	2.66	6.34	-0.393	1.85	3.94
O5 (BMDM2)	6.76	-0.468	1.83	2.05	6.36	-0.408	1.86	2.90
O6 (BMDM3)	6.77	-0.438	1.83	3.75	6.33	-0.361	1.85	6.08
O7 (BMDM3)	6.73	-0.432	1.82	4.12	6.35	-0.378	1.86	4.92
O8 (TPPO)	7.25	-0.352	1.96	8.75	6.84	-0.330	2.00	8.21

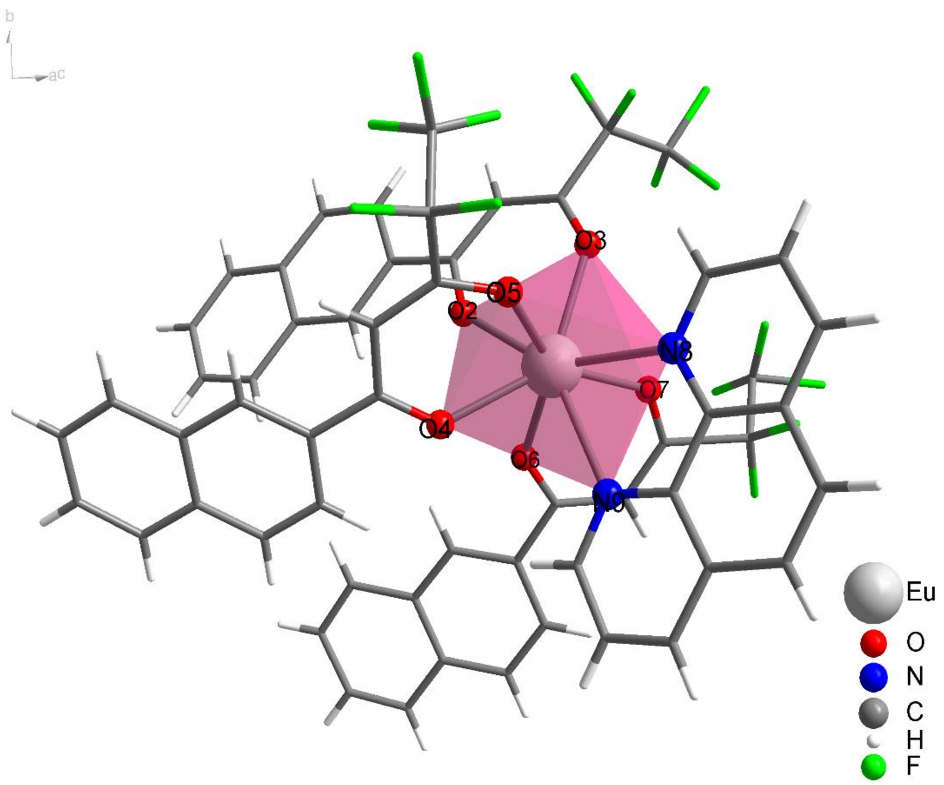

Figure S6. Perspective view of the crystallographic geometry of complex **Eu(PFNP)₃(PHEN)**, CSD code **QAMLEX**.

Table S6. Sparkle/RM1 and RM1 model for Eu(III) ZDO electronic densities *q* and electrophilic superdelocalizabilities *SE* for each atom directly coordinated to europium(III), in complex **Eu(PFNP)₃(PHEN)**, CSD code **QAMLEX**, at the crystallographic geometry, together with corresponding charge factors *g* and polarizabilities α from the fitting.

Ligand Atom	**Sparkle/RM1**<br><br>Q = 0.297 au^{-1}<br>D = 52.6 au^{-1}·Å^3<br>C = 26.7 Å^3<br>D/C = 2.03 au^{-1}				**RM1 model for Eu(III)**<br><br>Q = 0.315 au^{-1}<br>D = 59.8 au^{-1}·Å^3<br>C = 28.0 Å^3<br>D/C = 2.24 au^{-1}			
	q (au)	*SE* (au)	*g*	α (Å^3)	*q* (au)	*SE* (au)	*g*	α (Å^3)
O2 (PFNP1)	6.72	-0.409	1.99	5.15	6.34	-0.35	1.99	7.02
O3 (PFNP1)	6.71	-0.473	1.99	1.77	6.34	-0.44	1.99	1.79
O4 (PFNP2)	6.69	-0.455	1.99	2.76	6.35	-0.38	2.00	5.39
O5 (PFNP2)	6.73	-0.436	2.00	3.76	6.36	-0.40	2.00	4.22
O6 (PFNP3)	6.71	-0.485	1.99	1.18	6.35	-0.41	2.00	3.48
O7 (PFNP3)	6.72	-0.507	1.99	0.0054	6.35	-0.47	2.00	0.0053
N8 (PHEN)	5.48	-0.222	1.63	15.0	5.20	-0.22	1.64	15.0
N9 (PHEN)	5.48	-0.279	1.63	12.0	5.20	-0.25	1.64	12.9

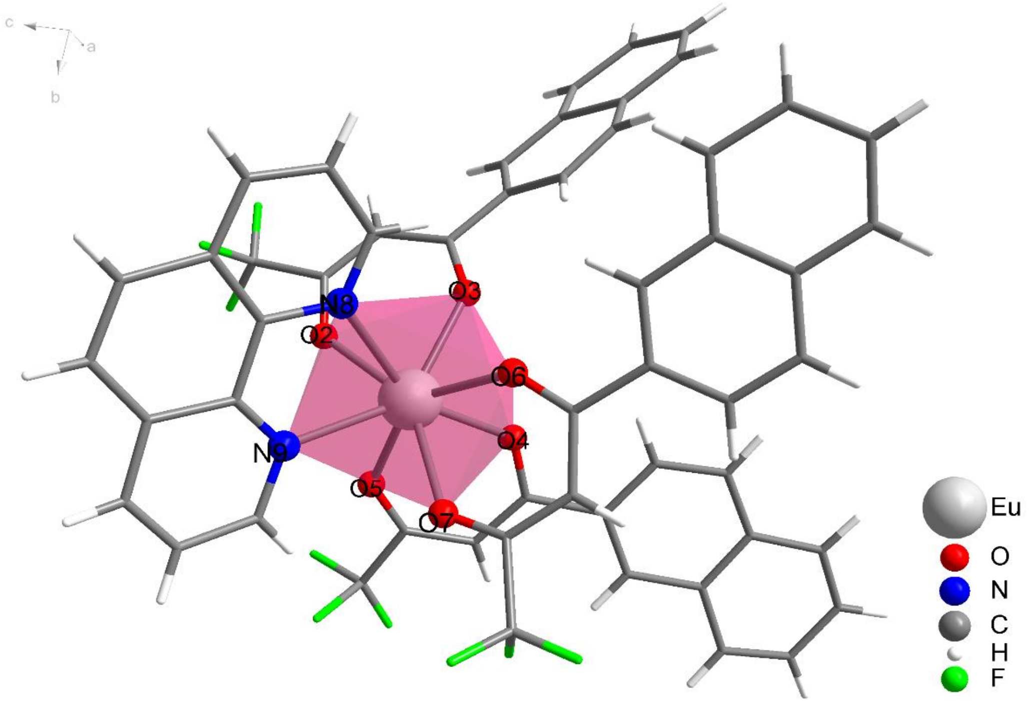

Figure S7. Perspective view of the crystallographic geometry of complex **Eu(TFNB)₃(PHEN)**, CSD code **QAMLIB**.

Table S7. Sparkle/RM1 and RM1 model for Eu(III) ZDO electronic densities *q* and electrophilic superdelocalizabilities *SE* for each atom directly coordinated to europium(III), in complex **Eu(TFNB)₃(PHEN)**, CSD code **QAMLIB**, at the crystallographic geometry, together with corresponding charge factors *g* and polarizabilities α from the fitting.

Ligand Atom	**Sparkle/RM1**<br><br>Q = 0.296 au^{-1}<br>D = 49.0 $au^{-1}\cdot Å^3$<br>C = 24.2 $Å^3$<br>D/C = 1.97 au^{-1}				**RM1 model for Eu(III)**<br><br>Q = 0.315 au^{-1}<br>D = 54.5 $au^{-1}\cdot Å^3$<br>C = 24.3 $Å^3$<br>D/C = 2.14 au^{-1}			
	q (au)	*SE* (au)	*g*	α ($Å^3$)	*q* (au)	*SE* (au)	*g*	α ($Å^3$)
O2 (TFNP1)	6.71	-0.493	1.99	0.0050	6.34	-0.446	2.00	0.0058
O3 (TFNP1)	6.72	-0.477	1.99	0.813	6.35	-0.398	2.00	2.64
O4 (TFNP2)	6.75	-0.324	2.00	8.28	6.36	-0.280	2.00	9.05
O5 (TFNP2)	6.69	-0.382	1.98	5.44	6.31	-0.357	1.98	4.88
O6 (TFNP3)	6.72	-0.479	1.99	0.695	6.35	-0.398	2.00	2.65
O7 (TFNP3)	6.68	-0.475	1.98	0.900	6.34	-0.432	1.99	0.79
N8 (PHEN)	5.50	-0.348	1.63	7.11	5.21	-0.301	1.64	7.94
N9 (PHEN)	5.47	-0.225	1.62	13.1	5.18	-0.216	1.63	12.5

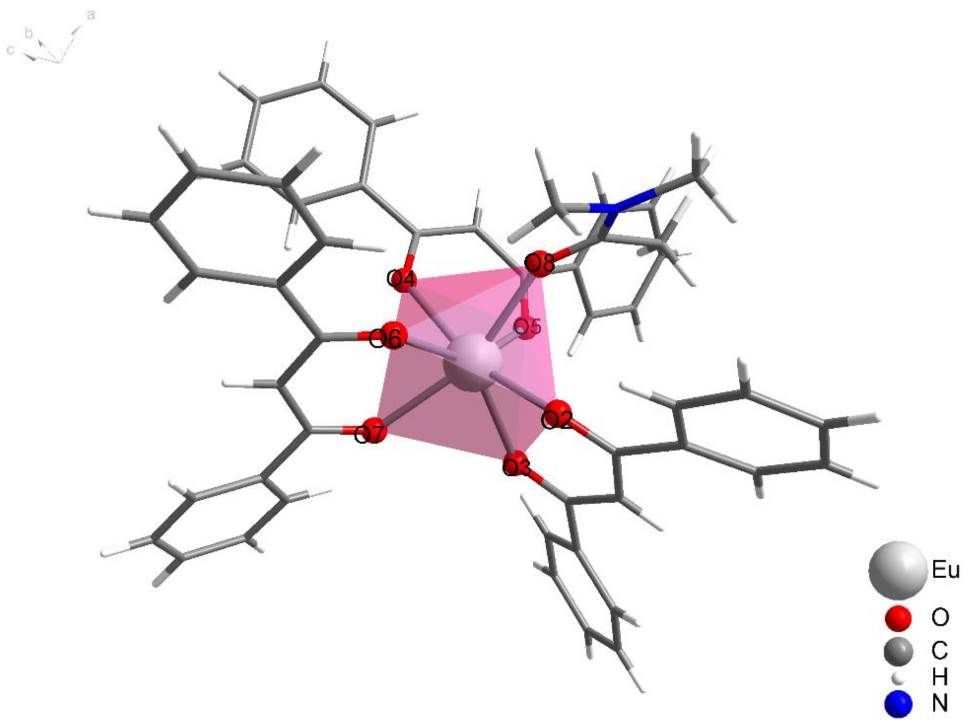

Figure S8. Perspective view of the crystallographic geometry of complex **Eu(DMB)₃(DMA)**, CSD code **RATKUU**.

Table S8. Sparkle/RM1 and RM1 model for Eu(III) ZDO electronic densities *q* and electrophilic superdelocalizabilities *SE* for each atom directly coordinated to europium(III), in complex **Eu(DMB)₃(DMA)**, CSD code **RATKUU**, at the crystallographic geometry, together with corresponding charge factors *g* and polarizabilities α from the fitting.

Ligand Atom	**Sparkle/RM1**<br>Q = 0.0546 au^{-1}<br>D = 60.2 au^{-1}·Å^3<br>C = 27.6 Å^3<br>D/C = 2.18 au^{-1}				**RM1 model for Eu(III)**<br>Q = 0.0550 au^{-1}<br>D = 46.7 au^{-1}·Å^3<br>C = 20.0 Å^3<br>D/C = 2.34 au^{-1}			
	q (au)	*SE* (au)	*g*	α (Å^3)	*q* (au)	*SE* (au)	*g*	α (Å^3)
O2 (DMB1)	6.77	-0.402	0.370	3.41	6.36	-0.350	0.350	3.63
O3 (DMB1)	6.72	-0.403	0.367	3.34	6.33	-0.335	0.348	4.36
O4 (DMB2)	6.74	-0.396	0.368	3.78	6.35	-0.362	0.349	3.08
O5 (DMB2)	6.75	-0.321	0.369	8.28	6.35	-0.279	0.349	6.97
O6 (DMB3)	6.77	-0.440	0.369	1.13	6.36	-0.390	0.349	1.76
O7 (DMB3)	6.76	-0.411	0.369	2.91	6.32	-0.341	0.348	4.04
O8 (DMA)	6.69	-0.431	0.365	1.71	6.36	-0.404	0.349	1.11

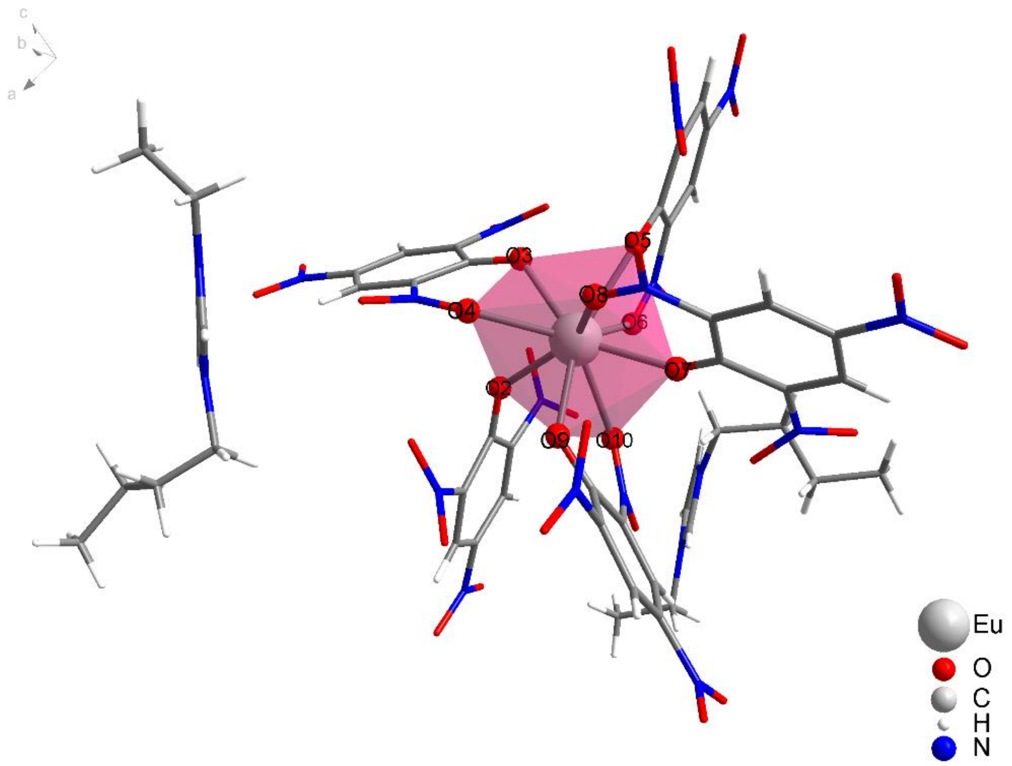

Figure S9. Perspective view of the crystallographic geometry of complex **(BEIm)$_2$[Eu(PIC)$_5$]**, CSD code **VENLEH**.

Table S9. Sparkle/RM1 and RM1 model for Eu(III) ZDO electronic densities *q* and electrophilic superdelocalizabilities *SE* for each atom directly coordinated to europium(III), in complex **(BEIm)$_2$[Eu(PIC)$_5$]**, CSD code **VENLEH**, at the crystallographic geometry, together with corresponding charge factors *g* and polarizabilities α from the fitting.

Ligand Atom	**Sparkle/RM1**<br><br>Q = 0.0372 au^{-1}<br>D = 17.6 au^{-1}·Å^3<br>C = 10.3 Å^3<br>D/C = 1.71 au^{-1}				**RM1 model for Eu(III)**<br><br>Q = 0.402 au^{-1}<br>D = 20.1 au^{-1}·Å^3<br>C = 10.6Å^3<br>D/C = 1.90 au^{-1}			
	q (au)	*SE* (au)	*g*	α (Å^3)	*q* (au)	*SE* (au)	*g*	α (Å^3)
O2 (PIC1)	6.72	-0.444	0.250	2.47	6.38	-0.419	0.257	2.17
O3 (PIC2)	6.65	-0.222	0.248	6.37	6.32	-0.213	0.254	6.30
O4 (PIC2)	6.47	-0.202	0.241	6.74	6.23	-0.202	0.251	6.52
O5 (PIC3)	6.67	-0.435	0.248	2.63	6.33	-0.387	0.255	2.80
O6 (PIC3)	6.56	-0.424	0.244	2.83	6.29	-0.380	0.253	2.95
O7 (PIC4)	6.72	-0.448	0.250	2.41	6.36	-0.379	0.256	2.96
O8 (PIC4)	6.54	-0.436	0.243	2.62	6.26	-0.408	0.252	2.38
O9 (PIC5)	6.69	-0.419	0.249	2.92	6.34	-0.392	0.255	2.71
O10 (PIC5)	6.50	-0.412	0.242	3.04	6.28	-0.378	0.253	2.99

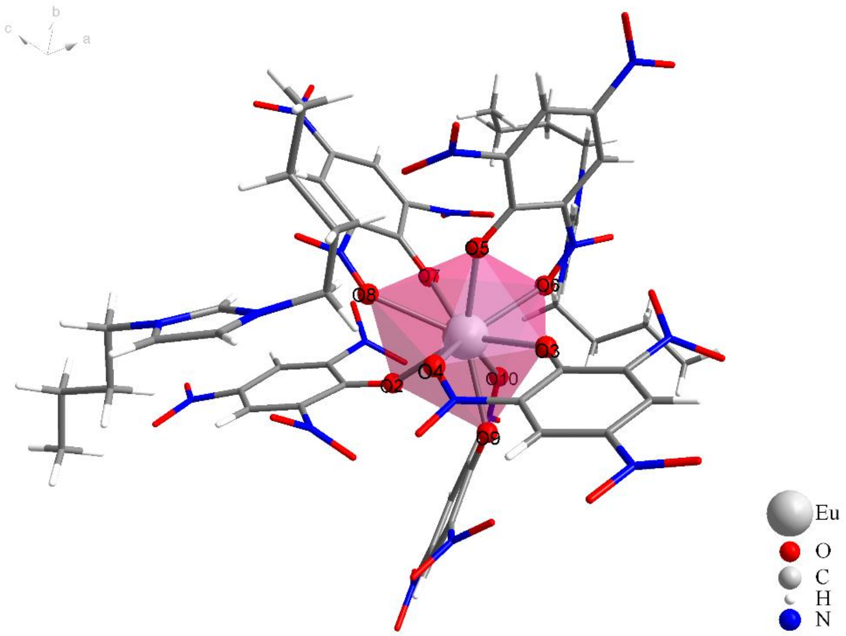

Figure S10. Perspective view of the crystallographic geometry of complex **(BBIm)$_2$[Eu(PIC)$_5$]**, CSD code **VENLIL**.

Table S10. Sparkle/RM1 and RM1 model for Eu(III) ZDO electronic densities q and electrophilic superdelocalizabilities *SE* for each atom directly coordinated to europium(III), in complex **(BBIm)$_2$[Eu(PIC)$_5$]**, CSD code **VENLIL**, at the crystallographic geometry, together with corresponding charge factors *g* and polarizabilities α from the fitting.

Ligand Atom	**Sparkle/RM1** Q = 0.0117 au^{-1} D = 22.2 au^{-1}·Å^3 C = 10.7Å^3 D/C = 2.08 au^{-1}				**RM1 model for Eu(III)** Q = 0.0239 au^{-1} D = 24.4 au^{-1}·Å^3 C = 10.7 Å^3 D/C = 2.27 au^{-1}			
	q (au)	*SE* (au)	*g*	α (Å^3)	*q* (au)	*SE* (au)	*g*	α (Å^3)
O2 (PIC1)	6.74	-0.431	0.119	1.11	6.38	-0.374	0.153	1.62
O3 (PIC2)	6.68	-0.432	0.118	1.09	6.34	-0.400	0.152	0.982
O4 (PIC2)	6.59	-0.437	0.117	0.973	6.29	-0.404	0.151	0.891
O5 (PIC3)	6.69	-0.274	0.119	4.60	6.35	-0.265	0.152	4.29
O6 (PIC3)	6.51	-0.209	0.115	6.05	6.26	-0.197	0.150	5.95
O7 (PIC4)	6.70	-0.287	0.119	4.31	6.35	-0.267	0.152	4.24
O8 (PIC4)	6.48	-0.290	0.115	4.25	6.28	-0.276	0.150	4.03
O9 (PIC5)	6.68	-0.345	0.118	3.03	6.34	-0.300	0.152	3.44
O10 (PIC5)	6.55	-0.285	0.116	4.35	6.28	-0.261	0.150	4.37

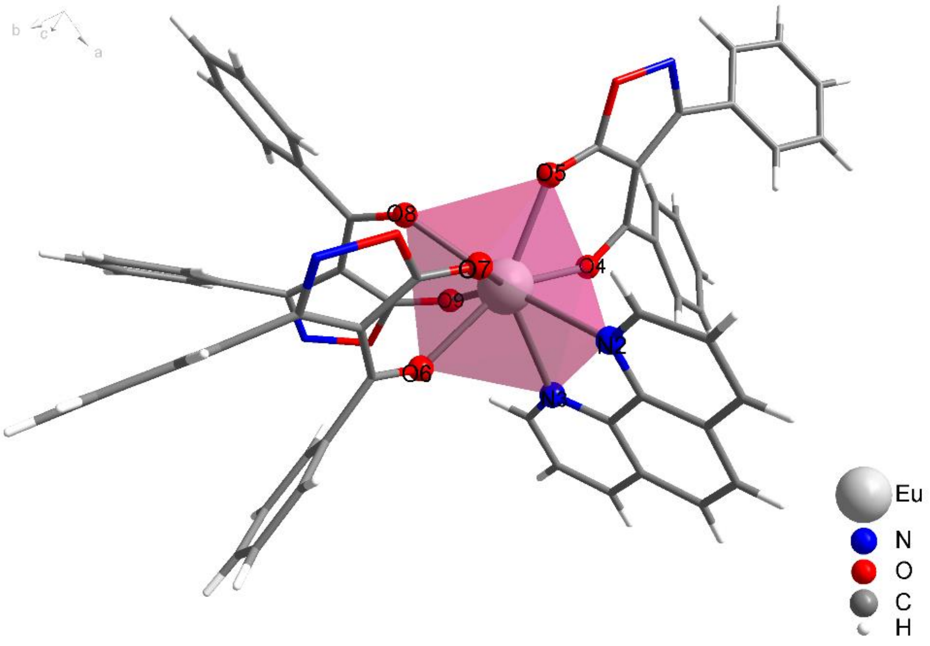

Figure S11. Perspective view of the crystallographic geometry of complex **Eu(PBI)$_3$(PHEN)**, CSD code **YETTOH**.

Table S11. Sparkle/RM1 and RM1 model for Eu(III) ZDO electronic densities q and electrophilic superdelocalizabilities *SE* for each atom directly coordinated to europium(III), in complex **Eu(PBI)$_3$(PHEN)**, CSD code **YETTOH**, at the crystallographic geometry, together with corresponding charge factors g and polarizabilities α from the fitting.

Ligand Atom	**Sparkle/RM1** Q = 0.297 au^{-1} D = 39.8 au^{-1}·Å^3 C = 18.6 Å^3 D/C = 2.13 au^{-1}				**RM1 model for Eu(III)** Q = 0.314 au^{-1} D = 39.1 au^{-1}·Å^3 C = 17.0 Å^3 D/C = 2.30 au^{-1}			
	q (au)	*SE* (au)	*g*	α (Å^3)	*q* (au)	*SE* (au)	*g*	α (Å^3)
N2 (PHEN)	5.50	-0.265	1.53	8.12	5.20	-0.250	1.63	7.20
N3 (PHEN)	5.46	-0.254	1.51	8.53	5.19	-0.248	1.63	7.28
O4 (PBI1)	6.72	-0.402	1.86	2.66	6.37	-0.364	2.00	2.72
O5 (PBI1)	6.69	-0.421	1.85	1.90	6.31	-0.348	1.98	3.35
O6 (PBI2)	6.72	-0.469	1.86	0.0058	6.37	-0.434	2.00	0.0050
O7 (PBI2)	6.69	-0.406	1.85	2.51	6.33	-0.349	1.98	3.31
O8 (PBI3)	6.66	-0.422	1.84	1.85	6.34	-0.363	1.99	2.79
O9 (PBI3)	6.72	-0.426	1.86	1.69	6.34	-0.379	1.99	2.16

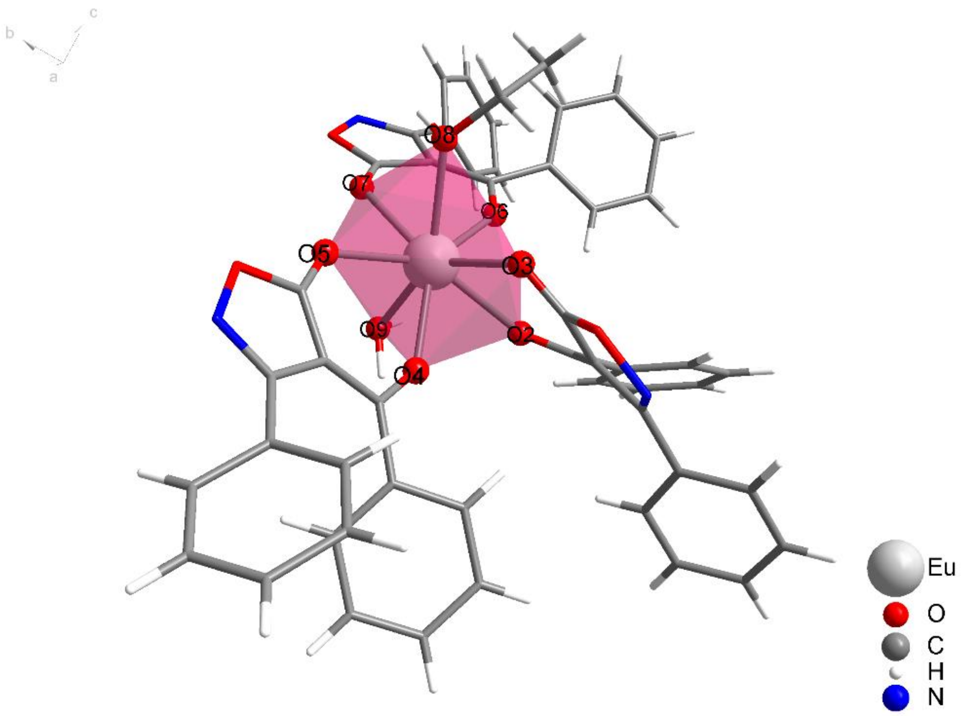

Figure S12. Perspective view of the crystallographic geometry of complex **Eu(PBI)$_3$(H$_2$O)(EtOH)**, CSD code **YETTUN**.

Table S12. Sparkle/RM1 and RM1 model for Eu(III) ZDO electronic densities q and electrophilic superdelocalizabilities *SE* for each atom directly coordinated to europium(III), in complex **Eu(PBI)$_3$(H$_2$O)(EtOH)**, CSD code **YETTUN**, at the crystallographic geometry, together with corresponding charge factors *g* and polarizabilities α from the fitting.

Ligand Atom	**Sparkle/RM1**<br><br>Q = 0.0729 au^{-1}<br>D = 29.0 au^{-1}·Å^3<br>C = 14.0 Å^3<br>D/C = 2.06 au^{-1}				**RM1 model for Eu(III)**<br><br>Q = 0.0855 au^{-1}<br>D = 32.6 au^{-1}·Å^3<br>C = 14.3 Å^3<br>D/C = 2.28 au^{-1}			
	q (au)	*SE* (au)	*g*	α (Å^3)	*q* (au)	*SE* (au)	*g*	α (Å^3)
O2 (PBI1)	6.68	-0.405	0.487	2.31	6.37	-0.365	0.545	2.40
O3 (PBI1)	6.72	-0.379	0.489	3.07	6.33	-0.324	0.542	3.72
O4 (PBI2)	6.75	-0.433	0.492	1.49	6.40	-0.359	0.548	2.57
O5 (PBI2)	6.70	-0.424	0.488	1.76	6.31	-0.385	0.540	1.75
O6 (PBI3)	6.75	-0.205	0.492	8.11	6.38	-0.194	0.545	7.94
O7 (PBI3)	6.67	-0.262	0.486	6.45	6.34	-0.252	0.542	6.08
O8 (EtOH)	6.51	-0.211	0.474	7.93	6.25	-0.207	0.535	7.52
O9 (H_2O)	6.55	-0.307	0.477	5.15	6.27	-0.294	0.536	4.68

Table S13. Fitted *Q*, *D*, and *C* values for all complexes studied. with electronic densities and electrophilic Superdelocalizabilities computed by **Sparkle/AM1** for Eu(III) at **Sparkle/AM1** fully optimized geometries, together with calculated and experimental Ω_λ values. The cells corresponding to geometries which led to unacceptable theoretical intensity parameters are painted gray.†

CSD code	*Q*	*D*	*C*	*D/C*	Ω_2^{calc}	$\Omega_2^{\exp}$	Ω_4^{calc}	$\Omega_4^{\exp}$	Ω_6^{calc}
854429[a]	0.0456	21.7	11.5	1.88	16.7	16.7	7.70	7.7	0.155
DEVHOC	0.156	10.2	10.7	0.947	40.9	40.9	17.5	17.5	0.374
EWOCOJ	0.144	61.1	28.4	2.15	19.0	19	2.87	2.6	0.438
GIPCAK	0.298	49.4	21.5	2.30	19.8	28.8	16.1	6.7	2.01
LOLXAN	0.0491	21.6	12.3	1.75	7.17	7.17	8.94	8.96	0.155
OTOYEC	0.269	0.128	4.59	0.0279	36.9	37.2	4.99	3.1	0.692
QAMLEX	0.298	46.0	22.9	2.01	45.4	46.3	11.4	7.8	2.08
QAMLIB	0.298	0.0002	7.17	0.0000	42.4	49	20.3	8.2	1.20
RATKUU	0.0621	56.4	26.5	2.13	51.0	51	6.72	6.7	0.223
VENLEH	0.0760	21.5	12.6	1.71	12.0	12	10.3	10.3	0.226
VENLIL	0.0550	12.2	7.59	1.61	9.61	9.6	9.20	9.2	0.153
YETTOH	0.210	28.2	13.8	2.04	15.5	15.7	2.52	1.53	0.907
YETTUN	0.107	25.7	13.3	1.93	16.5	16.5	14.3	14.29	0.345

†Units are: *Q* (au^{-1}); *D* ($au^{-1}.Å^3$); *C* ($Å^3$); Ω_λ ($10^{-20}cm^2$).

[a]Cambridge Crystallographic Data Centre deposited CSD entry.

Table S14. Fitted *Q*, *D*, and *C* values for all complexes studied. with electronic densities and electrophilic Superdelocalizabilities computed by **Sparkle/PM3** for Eu(III) at **Sparkle/PM3** fully optimized geometries, together with calculated and experimental Ω_λ values. The cells corresponding to geometries which led to unacceptable theoretical intensity parameters are painted gray.[†]

CSD code	*Q*	*D*	*C*	*D/C*	Ω_2^{calc}	$\Omega_2^{\exp}$	Ω_4^{calc}	$\Omega_4^{\exp}$	Ω_6^{calc}
854429[a]	0.289	0.0129	5.55	0.0023	16.2	16.7	8.57	7.7	0.840
DEVHOC	0.295	0.0003	8.14	0.0000	38.2	40.9	21.9	17.5	0.794
EWOCOJ	0.276	60.8	30.0	2.02	18.8	19	3.41	2.6	0.990
GIPCAK	0.296	0.0017	6.63	0.0003	24.1	28.8	13.9	6.7	0.955
LOLXAN	0.0694	26.2	15.3	1.71	7.15	7.17	8.96	8.96	0.164
OTOYEC	0.209	52.0	26.6	1.95	37.2	37.2	3.55	3.1	0.411
QAMLEX	0.295	50.8	25.9	1.96	44.6	46.3	10.1	7.8	1.42
QAMLIB	0.296	0.0054	8.80	0.0006	37.4	49	24.4	8.2	0.938
RATKUU	0.294	9.02	9.77	0.923	50.9	51	7.69	6.7	0.638
VENLEH	0.0025	0.0237	4.09	0.0058	12.3	12	9.87	10.3	0.0793
VENLIL	0.0017	0.347	3.92	0.0884	10.3	9.6	8.41	9.2	0.0682
YETTOH	0.124	31.7	14.9	2.13	15.6	15.66	1.66	1.53	0.222
YETTUN	0.0015	21.6	12.3	1.75	18.8	16.47	9.74	14.3	0.384

[†]Units are: *Q* (au^{-1}); *D* ($au^{-1}.Å^3$); *C* ($Å^3$); Ω_λ ($10^{-20}cm^2$).

[a]Cambridge Crystallographic Data Centre deposited CSD entry.

Table S15. Fitted Q, D, and C values for all complexes studied. with electronic densities and electrophilic Superdelocalizabilities computed by **Sparkle/PM6** for Eu(III) at **Sparkle/PM6** fully optimized geometries, together with calculated and experimental Ω_λ values. The cells corresponding to geometries which led to unacceptable theoretical intensity parameters are painted gray.[†]

CSD code	Q	D	C	D/C	Ω_2^{calc}	$\Omega_2^{\exp}$	Ω_4^{calc}	$\Omega_4^{\exp}$	Ω_6^{calc}
854429[a]	0.0728	20.1	11.7	1.72	16.7	16.7	7.70	7.7	0.145
DEVHOC	0.0015	10.4	9.93	1.05	42.6	40.9	10.1	17.5	0.733
EWOCOJ	0.290	0.0028	5.71	0.0005	13.6	19	9.39	2.6	1.22
GIPCAK	0.0830	26.5	14.1	1.89	28.8	28.8	6.70	6.7	0.274
LOLXAN	0.0542	16.0	10.7	1.50	7.16	7.17	8.96	8.96	0.146
OTOYEC	0.137	39.9	22.0	1.82	37.2	37.2	3.34	3.1	0.252
QAMLEX	0.273	47.3	23.9	1.98	46.0	46.3	9.29	7.8	1.49
QAMLIB	0.289	0.0069	7.94	0.0009	40.7	49	21.9	8.2	1.13
RATKUU	0.0019	23.3	15.1	1.55	51.0	51	6.45	6.7	0.291
VENLEH	0.0646	4.19	6.17	0.680	12.0	12	10.3	10.3	0.146
VENLIL	0.0015	0.655	3.73	0.176	10.4	9.6	8.19	9.2	0.147
YETTOH	0.153	26.7	13.5	1.98	15.6	15.66	1.97	1.53	0.457
YETTUN	0.0015	1.95	3.71	0.526	18.2	16.47	2.39	14.29	0.203

[†]Units are: Q (au^{-1}); D (au^{-1}.Å^3); C (Å^3); Ω_λ (10^{-20}cm^2).

[a]Cambridge Crystallographic Data Centre deposited CSD entry.

Table S16. Fitted *Q*, *D*, and *C* values for all complexes studied. with electronic densities and electrophilic Superdelocalizabilities computed by **Sparkle/PM7** for Eu(III) at **Sparkle/PM7** fully optimized geometries, together with calculated and experimental Ω_λ values. The cells corresponding to geometries which led to unacceptable theoretical intensity parameters are painted gray.†

CSD code	*Q*	*D*	*C*	*D/C*	Ω_2^{calc}	$\Omega_2^{\exp}$	Ω_4^{calc}	$\Omega_4^{\exp}$	Ω_6^{calc}
854429[a]	0.148	40.8	20.9	1.95	16.6	16.7	7.76	7.7	0.364
DEVHOC	0.144	17.4	14.3	1.22	40.9	40.9	17.5	17.5	0.442
EWOCOJ	0.195	32.3	17.6	1.83	18.8	19	3.44	2.6	0.707
GIPCAK	0.289	0.0112	5.76	0.0019	26.7	28.8	11.3	6.7	1.11
LOLXAN	0.0019	10.5	9.04	1.16	7.33	7.17	8.81	8.96	0.275
OTOYEC	0.0704	21.9	11.1	1.97	37.2	37.2	3.08	3.1	0.101
QAMLEX	0.289	0.0010	6.29	0.0002	43.2	46.3	15.4	7.8	1.24
QAMLIB	0.289	0.0020	7.06	0.0003	43.6	49	19.4	8.2	1.12
RATKUU	0.0978	16.9	12.8	1.33	51.0	51	6.63	6.7	0.203
VENLEH	0.0017	0.0166	3.66	0.0045	13.3	12	8.41	10.3	0.266
VENLIL	0.0017	0.0081	3.32	0.0024	10.2	9.6	8.48	9.2	0.106
YETTOH	0.190	29.1	14.8	1.96	15.5	15.66	2.27	1.53	0.696
YETTUN	0.0015	3.88	5.91	0.656	19.1	16.47	6.00	14.29	0.144

†Units are: *Q* (au^{-1}); *D* ($au^{-1}.Å^3$); *C* ($Å^3$); Ω_λ ($10^{-20}cm^2$).

[a]Cambridge Crystallographic Data Centre deposited CSD entry.
